# Efficacy and Safety of rAAV2-ND4 Treatment for Leber’s Hereditary Optic Neuropathy

**DOI:** 10.1038/srep21587

**Published:** 2016-02-19

**Authors:** Xing Wan, Han Pei, Min-jian Zhao, Shuo Yang, Wei-kun Hu, Heng He, Si-qi Ma, Ge Zhang, Xiao-yan Dong, Chen Chen, Dao-wen Wang, Bin Li

**Affiliations:** 1 Department of Ophthalmology, Tongji Hospital, Tongji Medical College, Huazhong University of Science and Technology, Wuhan, China; 2 Department of Oncology, Central Hospital, Ezhou City, China; 3 Department of Microbial and Biochemical Pharmacy, School of Pharmaceutical Sciences, Sun Yat-sen University, Guangzhou, China; 4 Beijing FivePlus Molecular Medicine Institute Co. Ltd., Beijing, China; 5 Center Genetic Diagnosis, Tongji Hospital, Tongji Medical College, Huazhong University of Science and Technology, Wuhan, China

## Abstract

Leber’s hereditary optic neuropathy (LHON) is a mitochondrially inherited disease leading to blindness. A mitochondrial DNA point mutation at the 11778 nucleotide site of the NADH dehydrogenase subunit 4 (*ND4*) gene is the most common cause. The aim of this study was to evaluate the efficacy and safety of a recombinant adeno-associated virus 2 (AAV2) carrying *ND4* (rAAV2-ND4) in LHON patients carrying the G11778A mutation. Nine patients were administered rAAV2-ND4 by intravitreal injection to one eye and then followed for 9 months. Ophthalmologic examinations of visual acuity, visual field, and optical coherence tomography were performed. Physical examinations included routine blood and urine. The visual acuity of the injected eyes of six patients improved by at least 0.3 log MAR after 9 months of follow-up. In these six patients, the visual field was enlarged but the retinal nerve fibre layer remained relatively stable. No other outcome measure was significantly changed. None of the nine patients had local or systemic adverse events related to the vector during the 9-month follow-up period. These findings support the feasible use of gene therapy for LHON.

Leber’s hereditary optic neuropathy (LHON) is a neurodegenerative mitochondrial disease that has been first described in 1871 by German ophthalmologist Theodore Leber^1^. The primary clinical symptoms are painless acute or sub-acute central vision loss, either simultaneous or successive in both eyes. The vast majority of patients have visual acuities of less than 2 log MAR (logarithm of the minimum angle of resolution) in both eyes^2^.

LHON is caused by point mutations in mitochondrial DNA (mtDNA). The three most common of these mutations (affecting up to 95% of patients) occur at nucleotide positions 11778, 14484, and 3460^3^. In particular, the G-to-A point mutation at 11778 (G11778A) in the NADH dehydrogenase subunit 4 complex I (*ND4*) gene of the electron transport chain (also known as Wallace mutation)^4^ is present in 60% of LHON patients worldwide^5^. We have previously shown that this mutation is present in as many as 87.9% of LHON patients in China alone^6^.

There is no definitely effective treatment available for LHON. It has been reported that idebenone, a short-chain derivative of coenzyme Q10, is a potential agent to treat LHON^7^, but its treatment efficiency is controversial. Idebenone and vitamin B12 therapy was reported to be effective in some patients^8^, but failed for others^9^,^10^. The latest studies have shown that the para-benzoquinone analogue of coenzyme Q10 and idebenone, EPI-743, holds promise with improved pharmacologic properties; there was a genuine improvement in several tested parameters of visual function^11^,^12^,^13^. Nevertheless, new methods in the treatment of LHON still need to be explored^14^.

One such alternative method is gene therapy^15^,^16^. However, *ND4* is an mtDNA- encoded gene, and transduced adeno-associated virus 2 (AAV2)-ND4 can only enter the nucleolus, not mitochondria. To circumvent this problem, in the present study, we used allotopic expression technology: we constructed a nuclear version of the mitochondrial *ND4* gene by recoding it in the nuclear genetic code based on the methods reported by Corral-Debrinski *et al*.^17^,^18^ and already successfully used by others^19^,^20^. In a preliminary clinical trial, the eyes with the worst vision of three patients were first treated with a single unilateral intravitreal injection of AAV2-ND4. No side effects were observed during the 9-month follow-up. Therefore, later, we administered the AAV2-ND4 gene therapy to six other patients. The findings reported herein provide guidance towards the use of gene therapy for LHON.

## Results

We enrolled nine diagnosed LHON patients in this prospective study. There were 7 male and 2 female patients with a mean age of 19.22 ± 11.57 years (Table 1). Best-corrected visual acuity (BCVA) of the nine patients showed no change from the time of enrolment to surgery. Ophthalmologic and systemic examinations were performed before surgery. Routine blood and urine tests; liver, kidney, and immune function tests; and CD3+, CD3+/CD4+ and CD3+/CD8+ cell counts were normal (Supplementary Fig. S1, Supplementary Fig. S2). The anti-AAV2 neutralizing antibody assay was negative (Supplementary Fig. S3).

Patients 1, 2, and 3 received gene therapy in August–September of 2011 as the first group of patients, and patients 4 to 9 received gene therapy in October–December of 2012 as the second group of patients. Intravitreal rAAV2-ND4 injection in these nine patients was successfully performed. No complications were observed during or after the procedure.

### Ophthalmologic examinations

**Figure 1:**
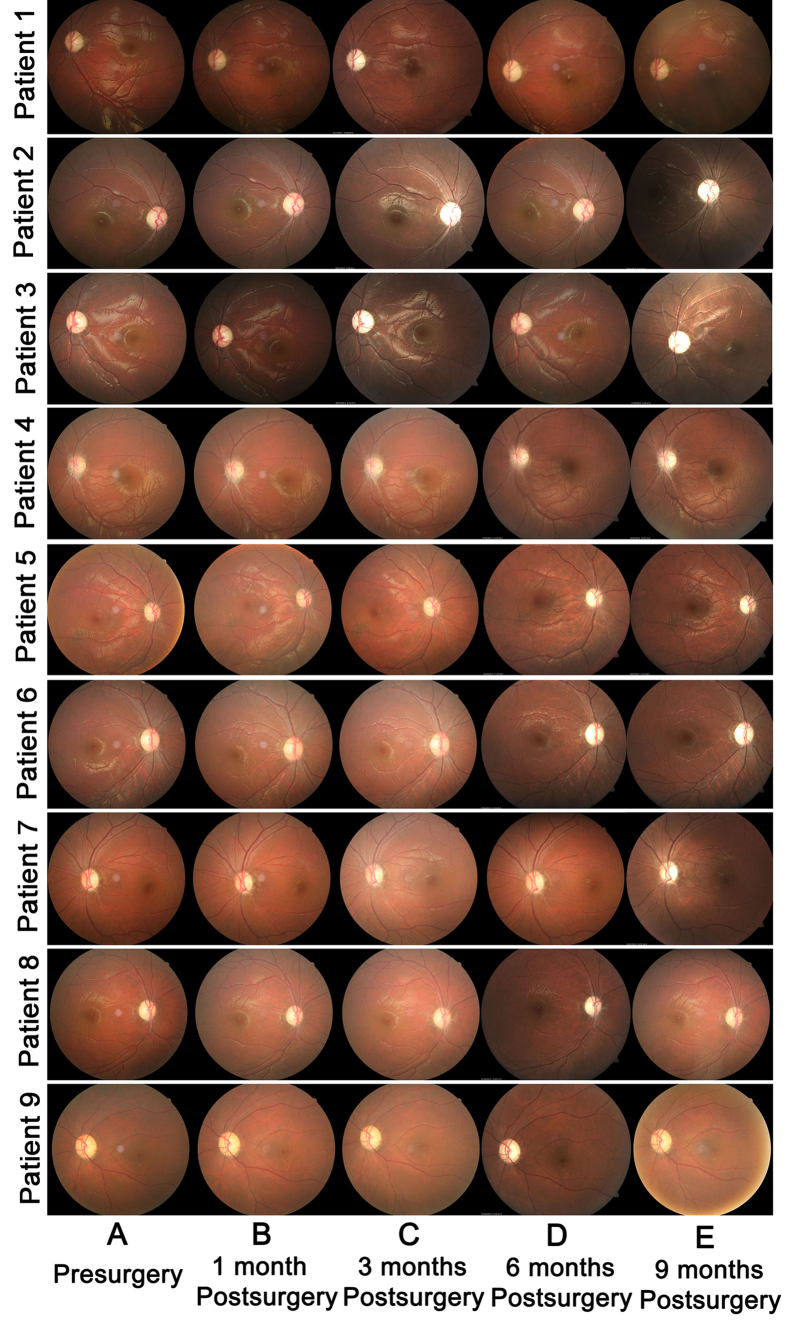
Retinal appearance and morphologic features before and after intravitreal injection of rAAV2- ND4 in the nine patients. (**A**) Representative 30° fundus photographs taken with a NIDEK Auto Fundus Camera show the disk and macula of injected eyes of patients before intravitreal injection. The retinal structure of the nine patients was normal. (**B** to **E**) 30° fundus photographs show the disk and macula of injected eyes of the nine patients at months 1, 3, 6, and 9 after intravitreal injection, respectively. No apparent retinal abnormality was found.

Ophthalmologic examinations were performed before treatment and 1, 3, 6, and 9 months after intravitreal injection. Intraocular pressure (IOP) and the anterior and posterior segments of the eye showed no change from before to after intravitreal injection (Fig. 1, Supplementary Fig. S4).

### Visual acuity

**Figure 2:**
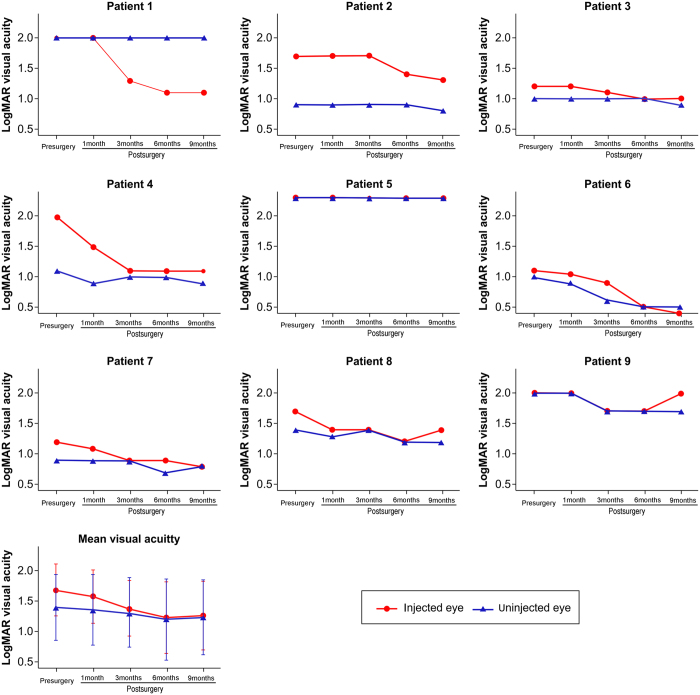
Visual acuity (log MAR) before and after intravitreal injection of rAAV2- ND4 in the nine patients. Mean best-corrected visual acuity (BCVA) values of injected and uninjected eyes before and 1, 3, 6 and 9 months after intravitreal injection are shown.

The visual acuity of six patients was improved significantly after 9 months of follow-up (the improvement of the BCVA was equal to or larger than 0.3 log MAR). Patients 1 and 4 improved their visual acuity from 2 to 1.1 log MAR, and patient 1 was able to read the newspaper headlines after treatment. Patient 2 had faced learning difficulties due to severe low vision, but his academic records improved markedly after treatment along with an improvement in visual acuity (from 1.7 to 1.3 log MAR). Patient 7 suffered from LHON for 17 years, but the visual acuity of the injected eye was also improved from 1.2 to 0.8 log MAR after treatment. Patient 6 (a 9-year-old girl) improved her visual acuity from 1.2 to 0.4 log MAR, and she has returned to school to study with her classmates. Interestingly, children with LHON have been reported to have an unfavourable prognosis^21^. Patient 8 improved his visual acuity as determined by ophthalmologic examinations, but the patient experienced no improvement (from 1.7 to 1.4 log MAR). It is worth noting that the visual acuity of the injected eye of patient 3 was little improved (from 1.2 to 1 log MAR), and the improvement did not reach the minimum of 0.3 log MAR. Patients 5 did not show any improvement of visual acuity, for unknown reasons. Mean visual acuity was 1.69 ± 0.43 before treatment. Mean BCVAs were 1.58 ± 0.44, 1.38 ± 0.46, 1.23 ± 0.60, and 1.27 ± 0.58 at 1, 3, 6, and 9 months after treatment, respectively (Fig. 2, Table 2).

### Vision field

**Figure 3:**
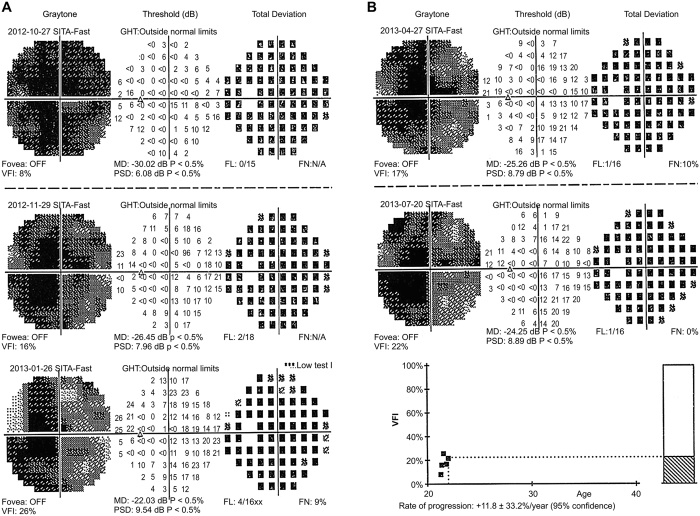
Visual field of the injected eye of patient 4 at the 9-month follow-up. The visual field of the injected eye of patient 4 was gradually improved at 1, 3, 6, and 9 months after treatment, and the visual field at 6 months after treatment was the best.

**Figure 4:**
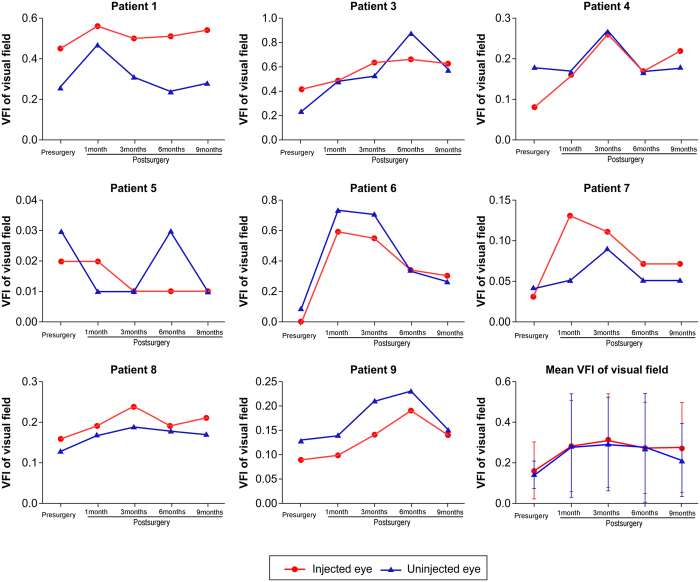
Visual field index (VFI) of injected and uninjected eyes of the nine patients. Mean VFI values of injected and uninjected eyes before and 1, 3, 6 and 9 months after intravitreal injection are shown. Eight patients had their VFI improved after treatment; the VFI of patient 5 was not improved.

**Figure 5:**
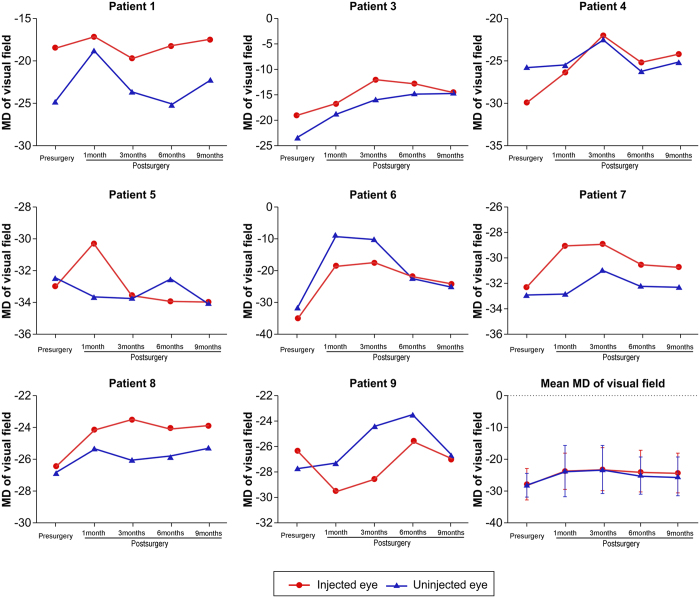
Mean defect (MD) of injected and uninjected eyes of the nine patients. Mean MD values of injected and uninjected eyes before and 1, 3, 6, and 9 months after intravitreal injection.

One month after intravitreal injection, patients 1, 4, and 6 reported an improvement in their visual field. Mean visual field index (VFI) of the injected eyes was 0.16 ± 0.18 before intravitreal injection, and 0.28 ± 0.23, 0.31 ± 0.23, 0.27 ± 0.22, and 0.27 ± 0.22 at months 1, 3, 6, and 9 after intravitreal injection, respectively (Figs 3 and 4). Mean defect (MD) of the injected eyes was improved at 6 and 9 months after intravitreal injection (Figs 3 and 5). Patient 2 refused the vision field test.

### VEP

VEP results showed the latency period of the P_100_ wave of the injected eyes of some patients was shortened (a P_100_ wave <105 ms is normal). At 6 months after intravitreal injection, the P_100_ amplitudes of the injected eyes were increased, but VEP results were unstable (Supplementary Fig. S5).

### OCT and electroretinography (ERG)

OCT revealed no significant change in retinal nerve fibre layer (RNFL) thickness of the nine patients in the superior, nasal, inferior, and temporal quadrants from before to after intravitreal injection (Supplementary Fig. S6).

Patients 2, 3, and 6 were too young to undergo ERG, and patients 5 and 8 refused to undergo ERG. Thus, the number of cases was too small, and we have no record of the ERG values.

### Systemic examinations

Systemic physical examinations were performed in the nine patients before and 1, 3, and 6 months after intravitreal injection. The results of routine blood and urine, and liver, kidney, and immune function tests of these nine patients were normal.

The detected values of CD3+/CD4+ and CD3+/CD8+ of patients 1, 2, and 9 were lower than the normal value (Supplementary Fig. S1). Six months later, CD3+/CD4+ and CD3+/CD8+ cell counts returned to normal in these patients.

The concentrations of serum AAV2, ND4, and interferon (IFN)-γ of the patients were determined by enzyme-linked immunosorbent assay (ELISA). Compared to pre-surgery values, the concentrations of serum AAV2, ND4, and IFN-γ were not significantly changed at months 1, 3, and 6 after treatment.

Before and 1 month after treatment, the AAV2-neutralizing antibody assay of patients 1 and 2 showed a difference between the AAV2 1:20 serum concentration and serum-free AAV2. However, 3 and 6 months after treatment, the results of the neutralizing antibody assay of the nine patients were not different between the AAV2 1:20 serum concentration and serum-free AAV2. Mean values were 0.93 ± 0.10, 0.86 ± 0.26, 0.92 ± 0.04, and 0.96 ± 0.07 before and 1, 3, and 6 months after intravitreal injection (Supplementary Fig. S3).

## Discussion

In the present study, we administered a single-dose intravitreal injection of rAAV2-ND4 to nine LHON patients. We found rAAV2-ND4 has the potential to treat LHON. No intravitreal injection-related adverse events or other complications occurred in these nine patients.

The safety of intravitreal rAAV-ND4 injection was ensured by prior standard screening and treatment. We chose for gene therapy those patients with a negative anti-AAV2 neutralizing antibody assay. The reason for such selection is that anti-AAV2 antibody may inhibit the expression of AAV2-ND4 in the retina, which could lead to an immune response. We medicated the patients with oral prednisolone one week before the treatment and for two months thereafter to avoid a potentially severe immune response induced by gene therapy. Six months after surgery, the results of the neutralizing antibody assay of these nine patients were all negative, suggesting that the systemic response to the single-dose intravitreal AAV2-ND4 injection was mild. It is reported that an AAV2-induced immune response is closely related to increases in CD8+ cells^22^,^23^. Therefore, we recruited patients with normal CD3+, CD3+/CD4+, and CD3+/CD8+ cell counts, in accordance with the practice of Bainbridge *et al*.^16^.

In our study, the visual acuity of the injected eyes improved mainly in the third and sixth months after treatment. In addition, we found that the eyesight of the injected eyes of patients 2 and 3 did not improve consistently 1 year after treatment.

LHON is caused by a point mutation in mtDNA, which leads to decreased NADH dehydrogenase activity in complex I of the mitochondrial respiratory chain, and thus a reduction in energy production by the mitochondria. Because optic nerve tissue requires much more energy than other tissues, it becomes severely damaged when energy production drops, and pathological changes in optic nerve cells and even atrophy can occur^24^,^25^,^26^. Given the results of the present study, it seems reasonable to suppose that gene therapy supplied normal ND4 to the mitochondria, and thus increased energy supply to the optic nerve. The microhabitat of the optic nerve tissues improved, making the recovery of optic function possible. The degree of improvement in optical function in these patients was primarily dependent on the degree of remaining optic nerve and RNFL function. Therefore, loss of vision in LHON patients was related to the condition of the optic nerve and not to the duration of sickness. For example, patient 7 suffered from LHON for 17 years, and her eyesight improved significantly after receiving treatment. Patient 4 suffered from LHON for 9 years, and his eyesight improved profoundly.

The present findings suggest that single-dose intravitreal rAAV2-ND4 injection can be safe and effective. One limitation of this study was the small sample size and the preliminary nature of the findings resulting thereof. A long-term, multicentre, and large-sample size study is necessary to confirm the potential of LHON gene therapy.

## Methods

### Construction of the rAAV plasmid

The original vector was produced and purified at Beijing FivePlus Molecular Medicine Institute Co. Ltd. (Beijing, China). The construction of the rAAV plasmid was performed in accordance with the method introduced by Bonnet *et al*.^17^ and Ellouze *et al*.^18^, and explained in detail by Pei *et al*.^27^. The promoter driving the expression of the *ND4* gene is the CMV promoter. Briefly, we first synthesized the DNA sequence coding for COX10 MTS-ND4–COX10 3′UTR in two parts: COX10 MTS-ND4 (sequence 1 comprising a mitochondrial-positioning signal in the COX10 5′ end+ COX10 MTS-ND4 CDS sequence) and COX10 3′UTR (sequence 2)^17^,^18^. The KpnI site was introduced into the 5′ end of sequence 1, and the SalI site into the 3′ end. The SalI site of the 5′ end was introduced into sequence 2; and the BamHI site into the 3′ end. The two sequences were then inserted into the plasmid pAAV2neo carrier, one at a time. The recombinant plasmid was examined by restriction enzyme digestion and named pAAV2neo-COX10-ND4-COX103′UTR. The COX10 MTS is 21 amino acids long. Thus, the fusion protein possesses 7 amino acids of the mature COX10 polypeptide (MAASPHTLSSRLL TGCVGGSVW YLE RRT).

Transfection of human embryonic kidney (HEK) 293 cells with pSNAV-ND4 was performed in 6-well plates using a Lipofectamine 2000 kit (Invitrogen). These cells were then transferred and cultured in a 110 × 480 mm^2^ flask (Wheaton, Wheaton, IL), and infected with HSV1-rc/ΔUL2 (Wu Jia He, Beijing, China; multiplicity of infection = 0.1) when the cell number reached 8 × 10^8^. The cells were then divided among 250 mL Fernbach culture flasks for further purification after 48 h of culture. The rAAV2-ND4 virus was purified and identified using reverse transcription (RT)-PCR. The rAAV2-ND4 titre (virus genomes/mL [vg/mL]) was detected using *in situ* hybridization with a digoxin-labelled cytomegalovirus probe. The final titre was 1 × 10^11^ vg/mL. In previous studies, we confirmed the safety and effectiveness of intravitreal injection of AAV2-ND4 *in vitro* and *in vivo* in laboratory animals^28^,^29^.

### Patients

Nine patients were included in this study. They were diagnosed with LHON carrying the G11778A mutation by genetic testing. The genetic tests were performed at Tongji Hospital Genetic Diagnosis Center (Wuhan, China). Patients did not suffer from any other diseases, nor did they take any medication within the previous year. Because 4% of LHON patients with the G11778A mutation recover spontaneously^30^, to ensure that improvements during the study were due to gene therapy alone, all the enrolees had been diagnosed for more than one year, and their BCVA had not changed within the previous year; patients with spontaneous visual recovery were excluded. Other inclusion criteria were: eyesight of both eyes <1.5 log MAR; age 8–60 years; negative anti-AAV2 neutralizing antibody assay; and ability to tolerate the gene therapy procedure, which includes local anaesthesia.

Exclusion criteria were as follows: patients wearing a cardiac pacemaker or suffering from severe heart, lung or kidney function failure, any haemorrhagic, acute infectious or mental disease, or high fever and those convalescing after heart surgery; patients suffering from chronic diseases such as diabetes and hypertension; pregnancy; patients participating in any other clinical study; patients with abnormal test results such as positive AAV2 humoral immune response (i.e. in an AAV2-neutralizing antibody assay, the percentage of inhibition of a 1:20 serum sample was significantly different from that of a no-antibody control) and abnormal human T lymphocyte subsets CD3+, CD3+/CD4+ and CD3+/CD8+ prior to gene therapy surgery. Gene therapy was administered to the eye with the poorer eyesight, or to the right eye if visual acuity was equivalent in the two eyes.

### Registration, informed consent and ethical approval

This study is registered with ClinicalTrials.gov (registration number, NCT01267422; registration date, December 2010). Our study was performed in accordance with the decision on the cancellation of non-administrative licensing examination and approval of the State Council of People’s Republic of China. The study was approved by the ethics committee of Ezhou Central Hospital and the methods used in this study were carried out in accordance with the approved guidelines. Each patient and their guardians signed informed consent forms. Institutional review board approval and written informed consent was obtained from each subject in accordance with the Declaration of Helsinki.

### Study design

Gene therapy consisted of injecting AAV2-ND4 into the vitreous cavity of the eye of LHON patients carrying the mtDNA 11778 mutation to induce the expression of ND4 protein at the diseased sites. This study was an open-label study.

### Systemic examinations

Before gene therapy, all patients underwent detailed systemic physical examinations. The systemic examination included routine blood and urine tests, and liver, kidney, and immune function tests. The venous blood of patients was collected and human T-lymphocyte subsets, namely CD3+ (normal range, 50–84%), CD3+/CD4+ (normal range, 27–51%) and CD3+/CD8+ (normal range, 15–44%), were counted. Anti-AAV2 neutralizing antibody level was analysed at the Department of Laboratory Medicine, Tongji Hospital, Huazhong University of Science and Technology (Supplementary Information). Serum level of AAV2, ND4, and IFN-γ was determined by ELISA (Supplementary Information).

Systemic examinations were repeated 1, 3, and 6 months after surgery.

### Ophthalmologic examinations

The primary endpoint of this study was defined as the recovery in visual acuity. The secondary endpoints were defined as the change in vision field, VEP, and OCT findings.

A complete ophthalmologic examination for visual acuity and IOP, an anterior segment examination by slit-lamp microscopy, and a fundus examination by ophthalmofundoscopy were performed in all patients. The slit-lamp microscopic and ophthalmoscopic examinations were conducted by the same medical doctor.

Visual acuity tests were based on the BCVA and were conducted by the same ophthalmologist. Eye charts were 2.5-m standard log MAR charts (Star Kang Medical Technology Co. Ltd., Wen Zhou, China) in which one line represented 0.3 log MAR units^11^,^31^. A change in the BCVA equal to or larger than 0.3 log MAR was considered significant. The patients were examined repeatedly three times every ten minutes to confirm their visual improvements, and the mean value was taken as the final visual acuity.

The tonometer used for determining IOP was a TOPCON-CT-80 Computerized Auto Tonometer (TOPCOCN, Tokyo, Japan). The mean value of three measurements was used.

For fundus (retinal) photography, we used the NIDEK Autofocus Fundus Camera, AFC-230 (Nidek, Tokyo, Japan).

A Humphrey field analyser (Carl Zeiss 740i, Carl Zeiss, Shanghai, China) was used for the visual field test. The testing procedure included the 30-2 central threshold test and SITA fast. The major recorded parameters were VFI and MD.

For VEP, the function of the optic nerve was examined after the value of the P_100_ wave was determined with a DV-100 (Shanghai Dikon Medical, Shanghai, China). The tested eyes were optically corrected, and the binocular viewing condition was adopted. The data acquisition and analysis were implemented with a connected computer.

Check-reversal amplitude was the difference between the first major positive peak near 100 ms (P_100_) and the preceding negative peak. The P_100_ amplitude was recorded. Main outcome measures included a latency period of the P_100_ wave and latency period of the reaction capacity of optic nerve conduction signals from the eye to the brain (the value <105 ms regarded as normal).

Retinal ganglion cell function was examined with a Roland Electronic GmbH electroretinogram (Keltern, Germany).

OCT was examined with a Spectralis® HRA+ OCT (Heidelberg Engineering, Heidelberg, Germany), and RNFL thickness of four quadrants (bottom, up, left, and right quadrants) of the retinal vessel was analysed. All data of OCT were automatically calculated using the existing software.

All examinations were conducted by the same technicians of the Ophthalmology Department of Tongji Hospital (Wuhan, China) before and 1, 3, 6, and 9 months after surgery.

### Oral prednisolone

Patients were given a 9-week course of oral prednisolone as described previously^15^,^16^. This regimen consisted of 0.5 mg/kg/day for the week before administration of the vector, 1 mg/kg/day during the first week afterward, 0.5 mg/kg/day for the second and third week, 0.25 mg/kg/day for the fourth and fifth week, and 0.125 mg/kg/day during the last three of the nine weeks. To prevent potentially severe adverse events during and after treatment, we established preventive measures for patients for whom emergencies were a possibility. Prednisolone is not efficient against LHON^32^.

### Intravitreal injection of rAAV2-ND4

The surgery was carried out in the operating room. After pupil enlargement, the patients were positioned supine on the operating table and 0.1% oxybuprocaine hydrochloride eye drops were used to anesthetize the appropriate eye. A 1-mL syringe was used, and the injecting pinhead was 30G. The injection site was 3.5 mm away from the temporal limbus. When the pinhead was visible through the pupil, rAAV2-ND4 was injected. Based on our previous experience *in vivo* in laboratory animals^28^ and on the available literature^15^,^16^, we selected a relatively lower dose than that used for gene therapy of congenital amaurosis. The dose was 5 × 10^9^ vg/0.05 mL for patients younger than 12 years old, and 1 × 10^10^ vg/0.05 mL for patients older than 12 years old. Children younger than 12 years old received half the dose for safety reasons.

## Additional Information

**How to cite this article**: Wan, X. *et al*. Efficacy and Safety of rAAV2-ND4 Treatment for Leber's Hereditary Optic Neuropathy. *Sci. Rep.* **6**, 21587; doi: 10.1038/srep21587 (2016).

## Supplementary Material

Supplementary Information

Supplementary Information 1

## Figures and Tables

**Table 1:** Clinical data of the nine LHON patients.

**Patient**	**Gender**	**Age (y)**		**Year of diagnosis**	**Treated eye**	**Date of gene therapy**	**Other notes**
		**Gene therapy**	**Emergence of LHON**				
First group							
1	Male	18	14	2009	Left	Aug. 2011	
2	Male	10	8	2010	Right	Sep. 2011	
3	Male	9	7	2010	Left	Sep. 2011	
Second group							
4	Male	21	13	2012	Left	Oct. 2012	Cousin of patient 3
5	Male	17	16	2012	Right	Oct. 2012	
6	Female	9	8	2012	Right	Nov. 2012	
7	Female	26	9	2010	Left	Nov. 2012	Aunt of patient 2
8	Male	17	13	2012	Right	Dec. 2012	
9	Male	46	43	2010	Left	Dec. 2012	

**Table 2:** Ophthalmologic examination results of the injected eyes before and 9 months after intravitreal injection.

**Patient**	**Eye**	**BCVA (log MAR)**		**VEP (P**_**100**_**, ms)**		**VEP (amp, nV)**		**MD of visual field (dB)**		**VFI of visual field (dB)**	
		**Before**	**After**	**Before**	**After**	**Before**	**After**	**Before**	**After**	**Before**	**After**
1	Left	2	1.1	169	115	1210	2870	−18.51	−17.53	45%	54%
2	Right	1.7	1.3	116	115	86.1	1910	Not recorded	Not recorded	Not recorded	Not recorded
3	Left	1.2	1	119	95	303	455	−19.47	−14.79	42%	63%
4	Left	2	1.1	139	154	1090	764	−30.02	−24.25	8%	22%
5	Right	2.3	2.3	144	122	2320	236	−33.01	−33.97	2%	1%
6	Right	1.1	0.4	116	130	152	2850	−34.92	−24.17	0%	30%
7	Left	1.2	0.8	125	130	146	3810	−32.41	−30.79	3%	7%
8	Right	1.7	1.4	128	124	763	1970	−26.45	−23.92	16%	21%
9	Left	2	2	132	151	618	1090	−29.36	−26.98	9%	14%
