# Supplementary material for: Efficacy and Safety of rAAV2-ND4 Treatment for Leber’s Hereditary Optic Neuropathy
Source: Sci Rep. 2016 Feb 19;6:21587. doi: 10.1038/srep21587 (PMC4759604; doi:10.1038/srep21587)
Supplement: Supplementary Information [file srep21587-s1.pdf]

Protocol

This supplement contains the following items:

1. Original protocol, final protocol, summary of changes. (highlighted in yellow track change in the final protocol).

2. Original statistical analysis plan, final statistical analysis plan, summary of changes.(highlighted in yellow track change in the final protocol).

CLINICAL TRIAL PROTOCOL

Intravitreal Injection of rAAV2-ND4 Treatment for Leber's Hereditary Optic Neuropathy

Protocol No.: 2010148
Version: 1

PRINCIPAL INVESTIGATOR Prof. Bin Li

Tongji Hospital Tongji Medical College, Huazhong University of Science and Technology Wuhan, Hubei, China

LIST OF ABBREVIATIONS AND DEFINITIONS

The following abbreviations and specialist terms are used in this study protocol:

**Abbreviation**	**Explanation**
AAV	Adeno-associated virus
BCVA	Best corrected visual acuity
CF	Counting fingers
ERG	Electroretinogram
HM	Hand movement
IOP	Intraocular pressure
LHON	Leber’s hereditary optic neuropathy
LogMAR	Logarithm of the minimum angle of resolution
MD	Mean defect
MtDNA	Mitochondrial DNA
ND4	NADH-ubiquinone oxidoreductase, subunit 4
OCT	Optical coherence tomography;
PSD	Pattern standard deviation;
RNFL	Retinal nerve fiber layer;
VEP	Visual evoked potential.
VFI	Visual field index.

STUDY CONTACT LIST

For questions regarding the conduct of this study, please contact:

Project Leader: Bin Li, PhD,MD
E-mail: **libin-12@163.com**
Telephone: +86 27 83663411
Mobile: 86-13638673626
Telefax: +86 27 83663411

Clinical Trial Manager: Xing Wan,MM

(Clinical investigator) E-mail: **422503912@qq.com** or **xingwan2@163.com**

Telephone: +86 27 83663223
Mobile: 86-13667254553
Telefax: +86 27 83663223

For reporting a serious adverse event, please contact:

Clinical Trial Manager: Shuo Yang
E-mail: **young_ophthalmology@foxmail.com**
Telephone: +86 27 83663410
Mobile: 86-13147179511
Telefax: +86 27 83663410

1.Background

Leber's hereditary optic neuropathy **(**LHON), which was first reported by the German scholar Theodore Leber in 1871, is a neurodegenerative genetic eye disease. It is caused by a gene mutation. The most common gene mutation is a mitochondrial mutation located at locus 11778. Most LHON patients are male. Onset of LHON usually occurs at 15 to 20 years of age. The primary clinical symptoms are acute or sub-acute painless eyesight degradation simultaneously or successively in both eyes, accompanied by central vision field loss and malfunction of chromatic vision.

There is no sufficient therapy for LHON currently available. Neurotrophic drugs and traditional Chinese medicine have both been used to treat LHON, There is no definitively effective treatment available for LHON. It has been reported that idebenone, a short chain derivative of coenzyme Q10, is a potential agent to treat LHON [1], but its treatment efficiency is controversial. Idebenone and vitamin B12 therapy is reported effective in some patients [2], but failed for others [3, 4]. The latest studies have shown that the para-benzoquinone analog of CoQ and idebenone, EPI-743, holds promise with improved pharmacologic properties; there was a genuine improvement in several tested parameters of visual function [5-7]. Although the preliminary results are encouraging, new methods in the treatment of LHON still need to be explored.

2 .Trial Objectives

The purpose of this study was to determine if, in humans, gene therapy for LHON caused by the mtDNA 11778 mutation would be associated with immediately obvious adverse events, and whether efficacy could be demonstrated.

3. Study population

Enrollees in the study were patients diagnosed with LHON who carried the mtDNA 11778 mutation. Patients did not suffer from any other diseases, and the best corrected visual acuity (BCVA) of both eyes was below 0.3. Because 4% of LHON patients

with the 11778 mutation recover spontaneously , to ensure that improvements during the study were due to gene therapy alone all the enrollees had been diagnosed for more than one year, and their BCVA had not changed within the previous year. Gene therapy was administered to the eye with the poorer eyesight. If the optic function of both eyes was casually identical, the right eye was chosen to receive gene therapy.

Criteria for inclusion

1) Patients carry the mitochondrial point mutation at locus 11778, which is consistent with the diagnostic criteria for LHON.
2) No apparent eye sight improvement in LHON patients or any other treatment within the past year.
3) Eyesight of both eyes is below 0.3.
4) Patients signed written informed consent.
5) Patients are between the ages of 8 and 60 years old and able to tolerate the gene therapy procedure which includes local anesthesia.
6) Patients are willing to follow the doctor's instructions and to consult the doctor at prescribed times.
7) Patient's physical examination results are all normal, including liver function, kidney function, routine blood test, routine urine test, complete immunological test, and humoral immune response.

Criteria of exclusion

1) Patients who are wearing a cardiac pacemaker, suffering from severe heart, lung or kidney function failure, various hemorrhagic diseases, acute infectious diseases, high fever, or convalescing after heart surgery or who are pregnant are excluded.
2) Patients who are participating in other clinical studies are excluded.
3) Patients who suffer from a diagnosed mental problem are excluded.
4) Patients who suffer from chronic diseases such as diabetes and hypertension are excluded.
5) Patients who show abnormal test results such as positive AAV2 humoral immune response (positive means that the AAV2 neutralizing antibody assay of patient was

significant different when comparing free serum with 1:20 serum concentrations) and abnormal human T lymphocyte subsets CD3+, CD3+/CD4+ and CD3+/CD8+ prior to gene therapy surgery are excluded.

4. Trial Design

The gene therapy consists of injecting adeno-associated virus 2-ND4 (AAV2-ND4) into the vitreous cavity of the eye of LHON patients caused by the mtDNA 11778 mutation to induce the expression of ND4 protein at the diseased sites ，

Ophthalmologic examination

Before gene therapy, all patients underwent detailed whole-body physical and ophthalmologic examinations. The ophthalmologic examinations consisted of BCVA, intraocular pressure (IOP), anterior segment examination by slit-lamp biomicroscopy, and direct opthalmoscopy of the fundus. Ocular fundus photographs, visual field test, OCT, VEP, and ERG were also performed.

Whole-body physical examinations

The whole-body examination included routine blood and urine, and liver, kidney, and immune function tests. The venous blood of patients was collected and cluster of differentiation (CD) human T lymphocyte subsets CD3+, CD3+ /CD4+, CD3+/CD8+, and the neutralizing antibody were analyzed by the Department of Laboratory Medicine, Tongji Hospital, Huazhong University of Science and Technology . In addition, we determined the concentration of serum AAV2, ND4, and interferon (IFN)-gamma of the patients via ELISA .

Oral prednisolone

Patients were given a 9-week course of oral prednisolone. This regimen consisted of 0. 5 mg/kg/day for the week before administration of the vector, 1 mg/kg/day during the first week afterward, 0.5 mg/kg/day for the second and third week, 0.25 mg/kg/day for the fourth and fifth week, and 0.125 mg/kg/day during the last three of the nine weeks. To prevent potentially severe adverse events during and after treatment, we

established preventative measures for patients for whom emergencies were a possibility.

Intravitreal injection

Injecting drug: AAV2/2-ND4

Injecting dose: 5×10^9 vg/0.05 mL

Procedure:The surgery was carried out in the operating room. Tropicamide eye drops (0.5%) were administrated 3 times, 30 minutes before surgery to enlarge the pupils. After pupil enlargement the patients lay supine on the operating table and 0.1% oxybuprocaine hydrochloride eye drops were used to anaesthetize the appropriate eye. The conjunctival sac was washed with 0.3% norfloxacin eye drops. A 1 mL syringe was used, and the injecting pinhead was 30G. The injection site was 3.5 mm away from the temporal limbus. The injecting pinhead was inserted into the conjunctiva, slid for 3 mm along the subconjunctiva, and then entered vertically into the vitreous body. When the pinhead was visible through the pupil, rAAV2-ND4 was injected. The needle was removed from the vitreous cavity. The injection site was pressed with a cotton swab after the intravitreal injection. The injected eye was covered with gauze, and the patient was allowed to lie on the operating table for 30 minutes.

Clinical observation

At months 1, 3, and 6 after surgery whole-body examinations were repeated, and at months 1, 3, 6, after surgery ophthalmologic examinations were repeated.

5. Risks and Benefits to participants and community

Risks

1) Accidents related to anesthesia, such as drug allergy and allergic shock.

2) The oculocardiac reflex may be induced when the eyeball is pressed and the patient’s life may be endangered;

3) The lens may be injured during the surgery, and cataract may be a complication;

4) Retinal detachment;

5) Postsurgery infection may affect the healing of the wound;

6) Postsurgical endophthalmitis may be a complication;

7) Eyesight may not improve after surgery;

8) Other potential risks include an immune rejection response, or impairment of liver function, kidney function and functions of other organs which may induce cancer and even endanger life;

9) Prednisone will be administrated orally for eight weeks in the present study, which often leads to upset stomach, increased blood pressure, rash, or urticaria.

10) Other potential unexpected or unpreventable complications.

Benefits to participants:

1、Accept the AAV2-ND4 Treatment free，

2、Ocular and systemic examination free at preoperative and postoperative.

3、Possible to improve vision.

6. Selection and Withdrawal of Subjects

Your participation in the study is completely dependent on your willingness. You can refuse to participate in the study, or, you can also withdraw from the study at any time in the course of research. Such a decision will not affect your relationship with the doctor, nor will it affect your medical or other benefits in any way.

To ensure your maximal benefits, if necessary the doctor or researcher may suspend your participation in the current study at any time during research.

If you withdraw from the current study for any reason, you probably will be asked questions relevant to your medication. If the doctor thinks it is necessary, you will be asked to accept a laboratory test and physical examination.

7. Treatment of Subjects

The treatment to be administered, including the dose, the dosing schedule, theroute/mode of administration, and the treatment periods, including follow-up:

See above.

Procedures to monitor subject compliance: All interventions will be conducted by study personnel; the only subject compliance required is to abide by scheduled visits.

8. Assessment of Efficacy

Visual acuity tests were conducted by an ophthalmologist. The eye chart was a 2.5-m standard ETDRS charts (Star Kang Medical Technology Co., Ltd. Wen Zhou China). We used two different eye charts to check patient's both eyes respectively. The patients were examined repeatedly to confirm their visual improvements, including tests at a different hospital performed by a completely uninformed oculist.

VEP, the function of the optic nerve was examined; primarily the value of the P_{100} wave was determined with a DV-100 (Shanghai Dikon Medical). The tested eyes were optically corrected, and the binocular viewing condition was adopted. The data acquisition and analysis were implemented with a connected computer. Check-reversal amplitude was the difference between the first major positive peak near 100 ms (P_{100}) and the preceding negative peak. The P_{100} amplitude was recorded. Main outcome measures included a latency period of the P_{100} wave and latency period of the reaction capacity of optic nerve conduction signals from the eye to the brain (the value <105 ms regarded as normal).

A Humphrey field analyzer (Carl Zeiss 740i, Carl Zeiss, Shanghai) was used for the visual field test. The testing procedure included the 30-2 central threshold test and SITA fast. Major record parameters were VFI, MD and PSD.

Electroretinogram(ERG): Retinal ganglion cell function was examined with a Roland Electronic GmbH electroretinogram (Keltern, Germany).

OCT was examined with a Spectralis® HRA + OCT (Heidelberg Engineering, Heidelberg, Germany), and the retinal nerve fiber layer (RNFL) thickness of four quadrants (bottom, up, left and right quadrants) of the retinal vessel was analyzed. All data of OCT were automatically calculated using the existing software.

The above examinations were conducted by two technicians of the Ophthalmology Department.

9. Assessment of Safety

The tonometer used for determining intraocular pressure was a TOPCON-CT-80 Computerized Auto Tonometer (TOPCOCN, Tokyo, Japan). The mean value of three measurements was used.

The fundus (retinal) photography used the NIDEK Autofocus Fundus Camera, AFC-230 (Nidek, Japan).

Whole-body physical examinations： Whole-body physical examinations consisted of routine blood and urine, and liver, kidney, and immune function tests analyzed by the Laboratory Department of Tongji Hospital. The specific examinations were those that are routine at Tongji Hospital.

Routine blood test included 24 items, such as white blood cell count and hemoglobin et al. The routine urine test included 24 items such as red blood cells, white blood cells, and bilirubin in urine et al. Liver and kidney function tests covered 11 items, including alanine aminotransferase, aspartate aminotransferase, and creatinine et al. The immune function test consisted of IgA, IgG, IgM, complement 3 and complement 4. The blood sample was sent to the Laboratory Department of our hospital. The tests for human T lymphocyte subsets CD3+, CD3+/CD4+ and CD3+/CD8+ were conducted by the Central Laboratory of Tongji Hospital. Venous blood from the patients was collected and sent to the Central Laboratory of Tongji Hospital and tested by the staff .

Neutralizing antibody assay： To detect neutralizing antibodies to AAV2, we incubated 1:20, 1:60, 1:180, 1:540, and 1:1620 mouse serum samples with 10^8 vg AAV2-GFP in 25 μL of PBS for 2 h at 4 °C. This mix was added to each well containing HEK293 cells grown in 6-well plates (to achieve a multiplicity of infection of 1000). The cells were grown at 37 ℃ in 5% CO_2, in Dulbecco’s Modified Eagle’s Medium (HyClone, Logan, UT) containing 5% FBS (HyClone). Green fluorescent protein expression was evaluated 48 h after infection by flow cytometry (BD Biosciences, NJ, USA). The percentage of inhibition was calculated with no-antibody control samples as a reference. Every experiment was repeated three times.

The levels of AAV2, IFN-γ and ND4 protein determined by ELISA assay： Serum samples from the nine patients were obtained preoperatively and 1, 3, and 6 months after intravitreal injection, and screened by ELISA for immunoreactivity to AAV2, IFN-γ and ND4 protein. The ELISA kits for AAV2, IFN-γ and ND4 were purchased from BlueGene Biotech. Shanghai, China. ELISA was performed according to the manufacturer's protocol.

10. Statistics METHODS

Determination of sample size

Since this is exploratory study, we will chose six patients of LHON to received a single-dose intravitreal injection of rAAV2-ND4.

Statistical analysis

A single statistical analysis will be performed at the end of the study. The data are expressed as mean ± standard error. Record the value of treated eyes, including: BCVA in logMAR , visual field record parameters were VFI, MD and PSD., VEP record parameters were the latency period and the amplitudes of the P100 wave , ERG record parameters were the latency period and the amplitudes of the P50 wave, thickness of the retinal nerve fiber layer, neutralizing antibody, and the concentrations of serum AAV2, ND4, and IFN-gamma were analyzed using the paired t-test. A probability (P) value of less than 0.05 was considered statistically significant.

Baseline

The data of treated yees at before treatment as baseline value, including BCVA, VFI, concentrations of serum AAV2, ND4, et al, These data were compared with month 1,3,6 after treatment of treated eyes.

11. ETHICS

Ethics review

The final study protocol, including the final version of the Subject Information and

Consent Forms, must be approved in writing by an Independent Ethics Committee (IEC). The Principle Investigator (Clinical Trial Manager) is responsible for informing the IEC of any serious adverse events (SAE) and amendment to the protocol as per regulatory requirement.

Ethical conduct of the study

The study will be performed in accordance with the ethical principles in the Declaration of Helsinki , and that are consistent with Good Clinical Practice and applicable regulatory requirements.

Subject information and consent

The Investigator will ensure that the subject is given full and adequate oral and written information about the nature, purpose, possible risk and benefit of the study. Subjects must also be notified that they are free to discontinue from the study at any time. The subject should be given the opportunity to ask questions and allowed time to consider the information provided. The subject's signed and dated informed consent must be obtained before conducting any study specific procedure. The investigator must store the original, signed Subject Informed Consent Form and a copy must be given to the subject.

11. STUDY TIME TABLE AND TERMINATION

First subject in	August 2011
Last patient out	March 2012
Clean File	March 2012
Study Report	April 2012

12. References

1. Y. Mashima, K. Kigasawa, M.Wakakura, et al. Do idebenone and vitamin therapy shorten the time to achieve visual recovery in Leber hereditary optic neuropathy? Journal of Neuro-Ophthalmology. 2000; 20: 166-170.
2. V. Carelli, P. Barboni, A. Zacchini, et al. Leber's hereditary optic neuropathy (LHON) with 14484/ND6 mutation in a north African patient. Journal of the Neurological Sciences. 1998;160:183-188.

3. Barnils N, Mesa E, Muñoz S, et al. Response to idebenone and multivitamin therapy in Leber’s hereditary optic neuropathy. Archivos de la Sociedad Espanola de Oftalmologia. 2007; 82: 377-380.
4. Angebault C, Gueguen N, Desquiret-Dumas V, et al. Idebenone increases mitochondrial complex I activity in fibroblasts from LHON patients while producing contradictory effects on respiration.BMC Res Notes. 2011, 22;4:557.
5. Klopstock T, Yu-Wai-Man P, Dimitriadis K, et al. A randomized placebo-controlled trial of idebenone in Leber's hereditary optic neuropathy. Brain. 2011; 134:2677-2686.
6. Carelli V, La Morgia C, Valentino ML, et al. Idebenone treatment in Leber's hereditary optic neuropathy. Brain. 2011; 134:e188.
7. Sadun AA, Chicani CF, Ross-Cisneros FN, et al. Effect of EPI-743 on the clinical course of the mitochondrial disease Leber hereditary optic neuropathy. Arch Neurol. 2012; 69:331-338.

APPENDIX 1

湖北省鄂州市中心医院医学伦理委员会临床试验审批表

〔2010〕 伦审字（148）号

项目名称	玻璃体腔注射 AAV-ND4 治疗 Leber 遗传性视神经病变的研究				
试验目的	1□ 2□ 3□ 4□ 5□ 6□ 7□ 8□ 9□ 10□ 11■				
申请单位	湖北省鄂州市中心医院		项目负责人	李斌　赵敏健	
意见	同意	作必要修改后同意	不同意	中止或暂停已批准的试验	
	9 票	0 票	0 票	0 票	

审评意见：

根据 ICH-GCP 的要求，本伦理会的组织和实施相对独立，本院伦理委员会的职责、人员组成、操作程序及记录遵循 ICH-GCP、中国的相关法律及法规。下面的（ √ ）的研究相关文件已审阅。

（ √ ）研究方案

（ √ ）病例报告表

（ √ ）知情同意书

经伦理委员会审议，意见如下：

1、入选标准中增加男女不限，提供选择儿童作为受试者的依据；

2、试验设计为自身前后对照，不存在试验组和对照组之分，

3、明确给药方法，给药次数

4、知情同意书的撰写应规范

湖北省鄂州市中心医院医务部　医学伦理委员会

主任签名：

备注：

1=I 期临床试验；2=II 期临床试验；3=III期临床试验；4=IV期临床试验；5=临床药代动力学试验；6=已有国家标准的药品的临床试验；7=进口药品临床试验；8=生物等效性；9=医疗器械临床验证；10=国际多中心临床试验；11=其他。

日期：2011 年 1 月 21 日

APPENDIX 2

DECLARATION OF HELSINKI

WORLD MEDICAL ASSOCIATION DECLARATION OF HELSINKI

Ethical Principles for Medical Research Involving Human Subjects

Adopted by the 18th WMA General Assembly, Helsinki, Finland, June 1964, and amended by the

29th WMA General Assembly, Tokyo, Japan, October 1975

35th WMA General Assembly, Venice, Italy, October 1983

41st WMA General Assembly, Hong Kong, September 1989

48th WMA General Assembly, Somerset West, Republic of South Africa, October 1996

and the 52nd WMA General Assembly, Edinburgh, Scotland, October 2000

Note of Clarification on Paragraph 29 added by the WMA General Assembly, Washington 2002

INTRODUCTION

The World Medical Association has developed the Declaration of Helsinki as a statement of ethical principles to provide guidance to physicians and other participants in medical research involving human subjects. Medical research involving human subjects includes research on identifiable human material or identifiable data.

It is the duty of the physician to promote and safeguard the health of the people. The physician's knowledge and conscience are dedicated to the fulfillment of this duty.

The Declaration of Geneva of the World Medical Association binds the physician with the words, "The health of my patient will be my first consideration," and the International Code of Medical Ethics declares that, "A physician shall act only in the patient's interest when providing medical care which might have the effect of weakening the physical and mental condition of the patient."

Medical progress is based on research, which ultimately must rest in part on experimentation involving human subjects.

In medical research on human subjects, considerations related to the well-being of the human subject should take precedence over the interests of science and society.

The primary purpose of medical research involving human subjects is to improve prophylactic, diagnostic and therapeutic procedures and the understanding of the aetiology and pathogenesis of disease. Even the best-proven prophylactic, diagnostic, and therapeutic methods must continuously be challenged through research for their effectiveness, efficiency, accessibility and quality.

In current medical practice and in medical research, most prophylactic, diagnostic and therapeutic procedures involve risks and burdens.

Medical research is subject to ethical standards that promote respect for all human beings and protect their health and rights. Some research populations are vulnerable and need special protection. The particular needs of the economically and medically disadvantaged must be recognized. Special attention is also required for those who cannot give or refuse consent for themselves, for those who may be subject to giving consent under duress, for those who will not benefit personally from the research and for those for whom the research is combined with care.

Research Investigators should be aware of the ethical, legal and regulatory requirements for research on human subjects in their own countries as well as applicable international requirements. No national ethical, legal or regulatory requirement should be allowed to reduce or eliminate any of the protections for human subjects set forth in this Declaration.

B. BASIC PRINCIPLES FOR ALL MEDICAL RESEARCH

10. It is the duty of the physician in medical research to protect the life, health, privacy, and dignity of the human subject.
11. Medical research involving human subjects must conform to generally accepted scientific principles, be based on a thorough knowledge of the scientific literature, other relevant sources of information, and on adequate laboratory and, where appropriate, animal experimentation.
12. Appropriate caution must be exercised in the conduct of research, which may affect the environment, and the welfare of animals used for research must be respected.
13. The design and performance of each experimental procedure involving human subjects should be clearly formulated in an experimental protocol. This protocol should be submitted for consideration, comment, guidance, and where appropriate, approval to a specially appointed ethical review committee, which must be independent of the investigator, the sponsor or any other kind of undue influence. This independent committee should be in conformity with the laws and regulations of the country in which the research experiment is performed. The committee has the right to monitor ongoing trials. The researcher has the obligation to provide monitoring information to the committee, especially any serious adverse events. The researcher should also submit to the committee, for review, information regarding funding, sponsors, institutional affiliations, other potential conflicts of interest and incentives for subjects.
14. The research protocol should always contain a statement of the ethical considerations involved and should indicate that there is compliance with the principles enunciated in this Declaration.
15. Medical research involving human subjects should be conducted only by scientifically qualified persons and under the supervision of a

clinically competent medical person. The responsibility for the human subject must always rest with a medically qualified person and never rest on the subject of the research, even though the subject has given consent.

16. Every medical research project involving human subjects should be preceded by careful assessment of predictable risks and burdens in comparison with foreseeable benefits to the subject or to others. This does not preclude the participation of healthy volunteers in medical research. The design of all studies should be publicly available.
17. Physicians should abstain from engaging in research projects involving human subjects unless they are confident that the risks involved have been adequately assessed and can be satisfactorily managed. Physicians should cease any investigation if the risks are found to outweigh the potential benefits or if there is conclusive proof of positive and beneficial results.
18. Medical research involving human subjects should only be conducted if the importance of the objective outweighs the inherent risks and burdens to the subject. This is especially important when the human subjects are healthy volunteers.
19. Medical research is only justified if there is a reasonable likelihood that the populations in which the research is carried out stand to benefit from the results of the research.
20. The subjects must be volunteers and informed participants in the research project.
21. The right of research subjects to safeguard their integrity must always be respected. Every precaution should be taken to respect the privacy of the subject, the confidentiality of the patient's information and to minimize the impact of the study on the subject's physical and mental integrity and on the personality of the subject.

22. In any research on human beings, each potential subject must be adequately informed of the aims, methods, sources of funding, any possible conflicts of interest, institutional affiliations of the researcher, the anticipated benefits and potential risks of the study and the discomfort it may entail. The subject should be informed of the right to abstain from participation in the study or to withdraw consent to participate at any time without reprisal. After ensuring that the subject has understood the information, the physician should then obtain the subject's freely given informed consent, preferably in writing. If the consent cannot be obtained in writing, the non-written consent must be formally documented and witnessed.
23. When obtaining informed consent for the research project the physician should be particularly cautious if the subject is in a dependent relationship with the physician or may consent under duress. In that case the informed consent should be obtained by a well-informed physician who is not engaged in the investigation and who is completely independent of this relationship.
24. For a research subject who is legally incompetent, physically or mentally incapable of giving consent or is a legally incompetent minor, the investigator must obtain informed consent from the legally authorized representative in accordance with applicable law. These groups should not be included in research unless the research is necessary to promote the health of the population represented and this research cannot instead be performed on legally competent persons.
25. When a subject deemed legally incompetent, such as a minor child, is able to give assent to decisions about participation in research, the investigator must obtain that assent in addition to the consent of the legally authorized representative.
26. Research on individuals from whom it is not possible to obtain consent, including proxy or advance consent, should be done only if the

physical/mental condition that prevents obtaining informed consent is a necessary characteristic of the research population. The specific reasons for involving research subjects with a condition that renders them unable to give informed consent should be stated in the experimental protocol for consideration and approval of the review committee. The protocol should state that consent to remain in the research should be obtained as soon as possible from the individual or a legally authorized surrogate.

27. Both authors and publishers have ethical obligations. In publication of the results of research, the investigators are obliged to preserve the accuracy of the results. Negative as well as positive results should be published or otherwise publicly available. Sources of funding, institutional affiliations and any possible conflicts of interest should be declared in the publication. Reports of experimentation not in accordance with the principles laid down in this Declaration should not be accepted for publication.

C. **ADDITIONAL PRINCIPLES FOR MEDICAL RESEARCH COMBINED WITH MEDICAL CARE**

28. The physician may combine medical research with medical care, only to the extent that the research is justified by its potential prophylactic, diagnostic or therapeutic value. When medical research is combined with medical care, additional standards apply to protect the patients who are research subjects.

29. The benefits, risks, burdens and effectiveness of a new method should be tested against those of the best current prophylactic, diagnostic, and therapeutic methods. This does not exclude the use of placebo, or no treatment, in studies where no proven prophylactic, diagnostic or therapeutic method exists. See footnote

30. At the conclusion of the study, every patient entered into the study should be assured of access to the best-proven prophylactic, diagnostic and therapeutic methods identified by the study.
31. The physician should fully inform the patient which aspects of the care are related to the research. The refusal of a patient to participate in a study must never interfere with the patient-physician relationship.
32. In the treatment of a patient, where proven prophylactic, diagnostic and therapeutic methods do not exist or have been ineffective, the physician, with informed consent from the patient, must be free to use unproven or new prophylactic, diagnostic and therapeutic measures, if in the physician's judgement it offers hope of saving life, re-establishing health or alleviating suffering. Where possible, these measures should be made the object of research, designed to evaluate their safety and efficacy. In all cases, new information should be recorded and, where appropriate, published. The other relevant guidelines of this Declaration should be followed.

FOOTNOTE:

NOTE OF CLARIFICATION ON PARAGRAPH 29 of the WMA DECLARATION OF HELSINKI

The WMA hereby reaffirms its position that extreme care must be taken in making use of a placebo-controlled trial and that in general this methodology should only be used in the absence of existing proven therapy. However, a placebo-controlled trial may be ethically acceptable, even if proven therapy is available, under the following circumstances:

- Where for compelling and scientifically sound methodological reasons its use is necessary to determine the efficacy or safety of a prophylactic, diagnostic or therapeutic method; or
- Where a prophylactic, diagnostic or therapeutic method is being investigated for a minor condition and the patients who receive placebo will not be subject to any additional risk of serious or irreversible harm.

All other provisions of the Declaration of Helsinki must be adhered to, especially the need for appropriate ethical and scientific review.

The Declaration of Helsinki (Document 17.C) is an official policy document of the World Medical Association, the global representative body for physicians. It was first adopted in 1964 (Helsinki, Finland) and revised in 1975 (Tokyo, Japan), 1983 (Venice, Italy), 1989 (Hong Kong), 1996 (Somerset-West, South Africa) and 2000 (Edinburgh, Scotland). Note of clarification on Paragraph 29 added by the WMA General Assembly, Washington 2002.

06.10.2002 09h00

APPENDIX 3a

PATIENT INFORMATION LEAFLET AND INFORMED CONSENT

Intravitreal Injection of rAAV2-ND4 Treatment for Leber's Hereditary Optic Neuropathy

Dear Patients:

We sincerely invite you to participate in this study. Please read the following information carefully before deciding whether to participate. It will help you understand the schedule, duration, benefits, risks, and discomfort inherent in the present study and the reason why this study is being performed. If you would like to participate, please consult the doctors for detailed information, and the doctors will help you. Alternatively, you can also discuss your options with your relatives and friends.

1. Background of Leber's hereditary optic neuropathy

Leber's hereditary optic neuropathy **(**LHON), which was first reported by the German scholar Theodore Leber in 1871, is a neurodegenerative genetic eye disease. It is caused by a gene mutation. The most common gene mutation is a mitochondrial mutation located at locus 11778. Most LHON patients are male. Onset of LHON usually occurs at 15 to 20 years of age. The primary clinical symptoms are acute or sub-acute painless eyesight degradation simultaneously or successively in both eyes, accompanied by central vision field loss and malfunction of chromatic vision.

There is no sufficient therapy for LHON currently available. Neurotrophic drugs and traditional Chinese medicine have both been used to treat LHON, but no positive potency was observed. In Japan, some researchers have suggested that the vasodilator idebenone could treat acute LHON. However, it is still in the clinical research stage and its efficacy is uncertain.

2. Objective of the study

The purpose of the current study was to assess the safety and potency of gene therapy

for LHON. The gene therapy consists of injecting adeno-associated virus 2-ND4 (AAV2-ND4) into the vitreous cavity of the eye of LHON patients to induce the expression of ND4 protein at the diseased sites

3. Criteria of inclusion and exclusion

Only patients of the Department of Ophthalmology of Tongji Hospital Affiliated with Tongji Medical School of Huazhong University of Science and Technology are to be enrolled in the study.

The criteria for inclusion are:

1) Patients carry the mitochondrial point mutation at locus 11778, which is consistent with the diagnostic criteria for LHON.
2) No apparent eye sight improvement in LHON patients or any other treatment within the past year.
3) Eyesight of both eyes is below 0.3.
4) Patients signed written informed consent.
5) Patients are between the ages of 8 and 60 years old and able to tolerate the gene therapy procedure which includes local anesthesia.
6) Patients are willing to follow the doctor's instructions and to consult the doctor at prescribed times.
7) Patient's physical examination results are all normal, including liver function, kidney function, routine blood test, routine urine test, complete immunological test, and humoral immune response.

The criteria of exclusion are follows:

1) Patients who are wearing a cardiac pacemaker, suffering from severe heart, lung or kidney function failure, various hemorrhagic diseases, acute infectious diseases, high fever, or convalescing after heart surgery or who are pregnant are excluded.
2) Patients who are participating in other clinical studies are excluded.
3) Patients who suffer from a diagnosed mental problem are excluded.
4) Patients who suffer from chronic diseases such as diabetes and hypertension are excluded.

5) Patients who show abnormal test results such as positive AAV2 humoral immune response (positive means that the AAV2 neutralizing antibody assay of patient was significant different when comparing free serum with 1:20 serum concentrations) and abnormal human T lymphocyte subsets CD3+, CD3+/CD4+ and CD3+/CD8+ prior to gene therapy surgery are excluded.

4. What is necessary for patient participation?

1) Before joining the present study, the doctor will inquire and record your medical history, and conduct examinations.

2) If you are qualified for inclusion, you will be asked whether you would like to participate voluntarily in the study, and to sign an informed consent form.

3) If you do not want to participate in the study, we will continue to treat you, as you wish.

4) If you would like to participate in the study, please expect to follow this procedure:

a. Sign the informed consent;

b. Allow pre-surgery examinations that include the eye and whole body;

c. Dilate the pupils of both eyes before surgery;

d. Submit to the operation, in which a single dose of approximately 5×10^9 vg AAV2-ND4 in 0.05 mL will be injected into the vitreous cavity of one eye;

e. Participate in the follow-up examinations, to be performed on days 1, 2, 3, and 7, and at the end of month 1, 3, and 6. The eye and whole-body examinations will be performed at every follow-up point and you can contact us at any time.

5. Potential adverse events, risks, discomfort and inconvenience

1) Accidents related to anesthesia, such as drug allergy and allergic shock.

2) The oculocardiac reflex may be induced when the eyeball is pressed and the patient’s life may be endangered;

3) The lens may be injured during the surgery, and cataract may be a complication;

4) Retinal detachment;

5) Postsurgery infection may affect the healing of the wound;

6) Postsurgical endophthalmitis may be a complication;

7) Eyesight may not improve after surgery;

8) Other potential risks include an immune rejection response, or impairment of liver function, kidney function and functions of other organs which may induce cancer and even endanger life;

9) Prednisone will be administrated orally for eight weeks in the present study, which often leads to upset stomach, increased blood pressure, rash, or urticaria.

10) Other potential unexpected or unpreventable complications.

6. Is personal information confidential?

Your medical record (patient history/case report form, laboratory test sheet) will be secured in the hospital. The doctor will record your laboratory test results in your patient history. The researcher, Hospital Ethics Committee, and Department of Drug Supervision and Administration are allowed to consult your medical record. Any public report relevant to the results of the present study will not reveal your personal information. Within the scope of legal permission, we try our best to keep secret your personal medical information.

7. How to get more information?

You can ask any question about the current study at any time, and get corresponding answers. During the study, if there is any important new information that might affect your willingness to participate, your doctor will inform you in time.

8. To participate in or to withdraw from the study is voluntary.

Your participation in the study is completely dependent on your willingness. You can refuse to participate in the study, or, you can also withdraw from the study at any time in the course of research. Such a decision will not affect your relationship with the doctor, nor will it affect your medical or other benefits in any way.

To ensure your maximal benefits, if necessary the doctor or researcher may suspend your participation in the current study at any time during research.

If you withdraw from the current study for any reason, you probably will be asked

questions relevant to your medication. If the doctor thinks it is necessary, you will be asked to accept a laboratory test and physical examination.

9. If you have any questions please contact Prof. Li Bin. Mobile phone number: 13638673626。

INFORMED CONSENT FORM

**Patient identification number: . . . . / . . . . .**	**Patient initials:**

Project title: Intravitreal Injection of rAAV2-ND4 Treatment for Leber's Hereditary Optic Neuropathy

Undertaker: Tongji Hospital, Tongji Medical College, Huazhong University of Science and Technology

By signing and dating this document,

- I confirm that I have had time to carefully read and understand the patient information sheet provided for this study.
- I confirm that I have had the opportunity to discuss the study and ask questions and I am satisfied with the answers and explanations that I have been provided.
- I give permission for my medical records to be reviewed by the Sponsor or designee, and/or representatives of the Medicines Control Council or Committee for Pharmaceutical Trials.
- I understand that my participation is voluntary and that I am free to withdraw at any time without giving any reason and without my medical care or legal rights being affected.
- I confirm that I have received a signed and dated copy of the Patient Information Sheet and Informed Consent Forms.

VOLUNTEER: ___________________ _________________

Name (capital letters) Signature Date

WITNESS: ___________________ *(where required)*

Name (capital letters) Signature Date

PERSON OBTAINING CONSENT: ___________________

Name (capital letters) Signature Date

CLINICAL INVESTIGATOR: ___________________ _________________

APPENDIX 3b
玻璃体腔注射 AAV2-ND4 治疗 Leber 遗传性视神经病变

知情同意书

亲爱的患者：

医生已经确诊您为 Leber 遗传性视神经病变。我们将邀请您参加这项研究。

在您决定是否参加这项研究之前，请尽可能仔细阅读以下内容。它可以帮助您了解该项研究以及为何要进行这项研究，研究的程序和期限，参加研究后可能给您带来的益处、风险和不适。如果您愿意，您也可以和您的亲属、朋友一起讨论，或者请医生给予解释，帮助您做出决定。

一、 研究背景和研究目的

1.1 疾病概况和治疗现况

Leber 遗传性视神经病变是一种主要累及黄斑乳头束纤维，导致视神经退行性变的遗传性疾病。由线粒体突变引起，11778 是最常见的位点。它是遗传性视神经病变的常见类型，由德国学者 Leber 于 1871 年首先报道。本病男性患者居多，常于 15-20 岁发病，临床主要表现为双眼同时或先后急性或亚急性无痛性视力减退，同时可伴有中心视野缺失及色觉障碍。

LHON 至今尚无有效的治疗方法。有日本研究者使用血管扩张剂艾地苯醌治疗急性期病例，尚在临床研究阶段，疗效并不确切。临床有使用神经营养药物治疗，但并无肯定的疗效，还有使用中医中药治疗，但未进行科学的临床试验验证。

1.2 本研究目的

本次研究的目的在于评价基因治疗 Leber 遗传性视神经病变的安全性和疗效，将重组腺相关病毒 2-ND4（AAV2-ND4）0.06ml 左右注射到患眼的玻璃体腔（单次），通过基因转染的方式在患眼病变部位表达正常的 ND4 蛋白，达到治疗疾病的目的。

1.3 研究参加单位和纳入患者例数

本研究的单位为华中科技大学同济医学院附属同济医院眼科，拟纳入 7 名患者，

入选标准为：

入选标准：

1. 符合 Leber 遗传性视神经病变诊断标准，并且为 11778 位点突变，近一年视力未提高者。
2. 双眼视力均在 0.3 一下。
3. 患者知情同意、自愿参加，男女不限。
4. 签署知情同意书。
5. 8 岁≤年龄≤60 岁，患者能耐受局麻手术。
6. 遵从医生的指示，能在规定的时间复诊。
7. 我们选择单眼单次玻璃体腔注射治疗，视力最差眼为注射眼，两眼视力相同则选择右眼作为注射眼。
8. 患者除了患有 Leber 遗传性视神经病变,其他检查均正常(肝肾功能、血常规、尿常规、免疫全套检测以及体液免疫反应等)

二、排除标准

1、佩带心脏起搏器者，心肺及肾功能严重衰弱，恶性肿瘤，各种出血性疾病，急性传感病，高热，高热性疾病，妇女妊娠期，心脏病手术后恢复期等。

2、正参加其它临床研究的病人。

3、有精神障碍的患者

4、患有糖尿病、高血压慢性疾病的患者。

5、孕妇或在哺乳期妇女。

6、术前检查项目异常，如 AAV2 体液免疫反应阳性，CD3/CD8 异常等。

三、如果参加研究将需要做什么？

1. 在您入选研究前，医生将询问、记录您的病史，并进行检查。

2.您是合格的纳入者，您可自愿参加研究，签署知情同意书。

3.如您不愿参加研究，我们将按您的意愿施治。

4. 若您自愿参加研究，将按以下步骤进行：

4.1. 签署知情同意书；

4.2. 进行术前检查，包括眼部和全身检查；

4.3. 术前准备；

4.4. 手术，单眼单次玻璃体腔注射 AAV2-ND4 0.05ml/5×10^9左右；

4.5. 术后定期检查：术后 1 天、2 天、3 天、7 天，1 月、3 月、半年。

4.6. 我们将长期追踪观察您的眼部和全身情况，您可以随时与我们联系。

四、参加研究可能的受益

1.将免费获得 AAV2-ND4 治疗

2.术前和术后免费进行眼部和全身检查。

3. 可能缓解疾病,提高视力。

五、参加研究可能的不良反应、风险和不适、不方便

1. 麻醉以外，药物过敏，过敏性休克，需进行抢救或暂停手术，严重情况可危及生命；
2. 压迫眼球诱发眼心反射发生危及生命；
3. 术中损伤晶状体，并发白内障；
4. 术中损伤视网膜，导致视网膜脱离；
5. 术后感染，影响伤口愈合；
6. 术后并发眼内炎；
7. 视力无提高；
8. 由于 AAV-ND4 是首次用于人体，因此它对于人体的毒副作用，目前尚未知晓，可能的风险包括损伤眼睛，严重的甚至失明，引起免疫反应，损伤患者肝肾及其他器官功能，引起肿瘤危及生命等等。
9. 本次研究将口服强的松 8 周，可能导致患者胃部不适，血压升高，皮疹或荨麻疹等。
10. 其他可能发生的无法预料或者不能防范的并发症等。

六、有关费用

1.将免费获得 AAV2-ND4 治疗

七、个人信息是保密的吗？

您的医疗记录（研究病历/CRF、化验单等）将完整地保存在您所就诊的医

院。医生会将化验检查结果记录在您的病历上。研究者、伦理委员会和药品监督管理部门将被允许查阅您的医疗记录。任何有关本项研究结果的公开报告将不会披露您的个人身份。我们将在法律允许的范围内，尽一切努力保护您个人医疗资料的隐私。

八、怎样获得更多的信息？

您可以在任何时间提出有关本项研究的任何问题，并得到相应的解答。

如果在研究过程中有任何重要的新信息，可能影响您继续参加研究的意愿时，您的医生将会及时通知您。

九、可以自愿选择参加研究和中途退出研究

是否参加研究完全取决于您的意愿。您可以拒绝参加此项研究，或在研究过程中的任何时间退出本研究，这都不会影响您和医生间的关系，都不会影响对您的医疗或有其他方面利益的损失。

出于对您的最大利益考虑，医生或研究者可能会在研究过程中随时中止您继续参加本项研究。

如果您因为任何原因从研究中退出，您可能被询问有关您使用试验药物的情况。如果医生认为需要，您被要求进行实验室检查和体格检查。

十．您有任何疑问可与李斌教授联系，联系电话 13638673626。

临床研究项目名称：

玻璃体腔注射 AAV-ND4 治疗 Leber 遗传性视神经病变的研究

课题承担单位: 华中科技大学同济医学院附属同济医院

同意声明

我已经阅读了上述有关本研究的介绍，而且有机会就此项研究与医生讨论并提出问题。我提出的所有问题都得到了满意的答复。

我知道参加本研究可能产生的风险和受益。我知晓参加研究是自愿的，我确认已有充足时间对此进行考虑，而且明白：

- 我可以随时向医生咨询更多的信息。
- 我可以随时退出本研究，而不会受到歧视或报复，医疗待遇与权益不会受到影响。

我同样清楚，如果我中途退出研究，特别是由于药物的原因使我退出研究时，我若将我的病情变化告诉医生，完成相应的体格检查和理化检查，这将对整个研究十分有利。

如果因病情变化我需要采取任何其他的药物治疗，我会在事先征求医生的意见，或在事后如实告诉医生。

我同意药品监督管理部门伦理委员会或申办者代表查阅我的研究资料。

我将获得一份经过签名并注明日期的知情同意书副本。

最后，我决定同意参加本项研究，并保证尽量遵从医嘱。

患者签名：______________ __ __ __ __ 年 __ __ 月 __ __ 日

患者监护人签名________________ __ __ __ __ 年 __ __ 月 __ __ 日

联系电话：______________

我确认已向患者解释了本实验的详细情况，包括其权力以及可能的受益和风险，并给其一份签署过的知情同意书副本。

医生签名：______________ __ __ __ __ 年 __ __ 月 __ __ 日

医生的工作电话：__________________

APPENDIX 4

PRINCIPAL INVESTIGATOR Prof. Bin Li

Published Literature in past 5 years

1. Han Pei, Xing Wan, Weikun Hu, xiaoyan Dong, **Li B***. Constructing and detecting a new rAAV2/2-ND4, Eye Science, 2013, 28:55-59.
2. Gao jing, Shi Hui,Pei Han, **Li B***, Comparison of Immunosuppressive Effects and ND4 Expression among Different Immunosuppressive Strategies following AAV2-ND4 Gene Treatment for Leber Hereditary Optic Neuropathy, Acta Medicinae Universitatis Scientiae et Technologiae Huazhong, 2013,2, 187-191.
3. Cui G, Ding H, Xu Y, **Li B***, Wang DW*****. Applications of the method of high resolution melting analysis for diagnosis of Leber's disease and the three primary mutation spectrum of LHON in the Han Chinese population. Gene. Jan 1;512(1):108-12, 2013.
4. Shi H, Gao J, Pei H, Liu R, Hu WK, Wan X, Li T, **Li B***. AAV-mediated gene delivery of the human ND4 complex I subunit in rabbit eyes. Clin Experiment Ophthalmol. Dec;40 (9) : 888- 94, 2012.
5. Wei-Kun Hu,Rong Liu, Han Pei, **Bin Li ***, Endoplasmic Reticulum Stress-Related Factors Protect against Diabetic Retinopathy ， Experimental Diabetes Research，2012，Epub 2011 Dec.
6. Shu Yan, Cui Zheng, Zhi-qi Chen, Rong Liu, Gui-gang Li, Wei-kun Hu, Han Pei, **Bin Li ***，Expression of Endoplasmic Reticulum Stress-Related Factors in the Retinas of Diabetic Rats，Experimental Diabetes Research , ID 743780, 11 pages, Epub 2011 Aug 28，2012.
7. Hong Yang, Rong Liu, Zheng Cui, Zhi-qi Chen, Shu Yan, Han Pei, **Bin Li***，Functional characterization of 58-kilodalton inhibitor of protein kinase in protecting against diabetic retinopathy via the endoplasmic reticulum stress pathway，Molecular Vision,17:78-84, 2011;

8. Nan Xiang, Min-Jian Zhao, Xin-Yu Li, Hai-Hong Zheng, Gui-Gang Li, **Li B***，Redundant Mechanisms for Vascular Growth Factors in Retinopathy of Prematurity in vitro. Ophthalmic Res.19;45(2):92-101, 2011。
9. **Li B***, Wang HS, Li GG, Zhao MJ, Zhao MH., The role of endoplasmic reticulum stress in the early stage of diabetic retinopathy. Acta Diabetol. 48(2):103-11, 2011。
10. **Bin Li**, Hai-Qing Zhang, Yu Shi, Yun-Bing Min,Shao-Fen Lin, Kai-Li Wu,Jie Hu, Shi-Bo Tang, Overexpression of nuclear transport factor 2 may protect against diabetic retinopathy, Molecular Vision , 15:861-869,2009.
11. **Bin Li***, Dong Li, Gui-gang Li, Hao-wen Wang, Ai-xia Yu , $P58^{IPK}$ inhibition of endoplasmic reticulum stress in human retinal capillary endothelial cells in vitro, Molecular Vision,4:1122- 1128, 2008.
12. **Bin Li*,** Wei Yin, Xun Hong, Yu Shi, Hong-Sheng Wang, Shao-Fen Lin, and Shi-Bo Tang*, Remodeling Retinal Neovascularization by ALK1 Gene Transfection In Vitro, IOVS, 49: 4553-4560, 2008.

CLINICAL TRIAL PROTOCOL

Intravitreal Injection of rAAV2-ND4 Treatment for Leber's Hereditary Optic Neuropathy

Protocol No.: 2010148
Version: 2

PRINCIPAL INVESTIGATOR Prof. Bin Li

Tongji Hospital Tongji Medical College, Huazhong University of Science and Technology Wuhan, Hubei, China

LIST OF ABBREVIATIONS AND DEFINITIONS

The following abbreviations and specialist terms are used in this study protocol:

Abbreviation	Explanation
AAV	Adeno-associated virus
BCVA	Best corrected visual acuity
CF	Counting fingers
ERG	Electroretinogram
HM	Hand movement
IOP	Intraocular pressure
LHON	Leber's hereditary optic neuropathy
LogMAR	Logarithm of the minimum angle of resolution
MD	Mean defect
MtDNA	Mitochondrial DNA
ND4	NADH-ubiquinone oxidoreductase, subunit 4
OCT	Optical coherence tomography;
PSD	Pattern standard deviation;
RNFL	Retinal nerve fiber layer;
VEP	Visual evoked potential.
VFI	Visual field index.

STUDY CONTACT LIST

For questions regarding the conduct of this study, please contact:

Project Leader: Bin Li, PhD,MD
E-mail: **libin-12@163.com**
Telephone: +86 27 83663411
Mobile: 86-13638673626
Telefax: +86 27 83663411

Clinical Trial Manager: Xing Wan,MM
(Clinical investigator) E-mail: **422503912@qq.com** or **xingwan2@163.com**
Telephone: +86 27 83663223
Mobile: 86-13667254553
Telefax: +86 27 83663223

For reporting a serious adverse event, please contact:

Clinical Trial Manager: Shuo Yang
E-mail: **young_ophthalmology@foxmail.com**
Telephone: +86 27 83663410
Mobile: 86-13147179511
Telefax: +86 27 83663410

1.Background

Leber's hereditary optic neuropathy **(**LHON), which was first reported by the German scholar Theodore Leber in 1871, is a neurodegenerative genetic eye disease. It is caused by a gene mutation. The most common gene mutation is a mitochondrial mutation located at locus 11778. Most LHON patients are male. Onset of LHON usually occurs at 15 to 20 years of age. The primary clinical symptoms are acute or sub-acute painless eyesight degradation simultaneously or successively in both eyes, accompanied by central vision field loss and malfunction of chromatic vision.

There is no sufficient therapy for LHON currently available. Neurotrophic drugs and traditional Chinese medicine have both been used to treat LHON, There is no definitively effective treatment available for LHON. It has been reported that idebenone, a short chain derivative of coenzyme Q10, is a potential agent to treat LHON [1], but its treatment efficiency is controversial. Idebenone and vitamin B12 therapy is reported effective in some patients [2], but failed for others [3, 4]. The latest studies have shown that the para-benzoquinone analog of CoQ and idebenone, EPI-743, holds promise with improved pharmacologic properties; there was a genuine improvement in several tested parameters of visual function [5-7]. Although the preliminary results are encouraging, new methods in the treatment of LHON still need to be explored.

2 .Trial Objectives

The purpose of this study was to determine if, in humans, gene therapy for LHON caused by the mtDNA 11778 mutation would be associated with immediately obvious adverse events, and whether efficacy could be demonstrated.

3. Study population

Enrollees in the study were patients diagnosed with LHON who carried the mtDNA 11778 mutation. Patients did not suffer from any other diseases, and the best corrected visual acuity (BCVA) of both eyes was below 0.3. Because 4% of LHON patients

with the 11778 mutation recover spontaneously , to ensure that improvements during the study were due to gene therapy alone all the enrollees had been diagnosed for more than one year, and their BCVA had not changed within the previous year. Gene therapy was administered to the eye with the poorer eyesight. If the optic function of both eyes was casually identical, the right eye was chosen to receive gene therapy.

Criteria for inclusion

1) Patients carry the mitochondrial point mutation at locus 11778, which is consistent with the diagnostic criteria for LHON.
2) No apparent eye sight improvement in LHON patients or any other treatment within the past year.
3) Eyesight of both eyes is below 0.3.
4) Patients signed written informed consent.
5) Patients are between the ages of 8 and 60 years old and able to tolerate the gene therapy procedure which includes local anesthesia.
6) Patients are willing to follow the doctor’s instructions and to consult the doctor at prescribed times.
7) Patient’s physical examination results are all normal, including liver function, kidney function, routine blood test, routine urine test, complete immunological test, and humoral immune response.

Criteria of exclusion

1) Patients who are wearing a cardiac pacemaker, suffering from severe heart, lung or kidney function failure, various hemorrhagic diseases, acute infectious diseases, high fever, or convalescing after heart surgery or who are pregnant are excluded.
2) Patients who are participating in other clinical studies are excluded.
3) Patients who suffer from a diagnosed mental problem are excluded.
4) Patients who suffer from chronic diseases such as diabetes and hypertension are excluded.
5) Patients who show abnormal test results such as positive AAV2 humoral immune response (positive means that the AAV2 neutralizing antibody assay of patient was

significant different when comparing free serum with 1:20 serum concentrations) and abnormal human T lymphocyte subsets CD3+, CD3+/CD4+ and CD3+/CD8+ prior to gene therapy surgery are excluded.

4. Trial Design

The gene therapy consists of injecting adeno-associated virus 2-ND4 (AAV2-ND4) into the vitreous cavity of the eye of LHON patients caused by the mtDNA 11778 mutation to induce the expression of ND4 protein at the diseased sites ，

Ophthalmologic examination

Before gene therapy, all patients underwent detailed whole-body physical and ophthalmologic examinations. The ophthalmologic examinations consisted of BCVA, intraocular pressure (IOP), anterior segment examination by slit-lamp biomicroscopy, and direct opthalmoscopy of the fundus. Ocular fundus photographs, visual field test, OCT, VEP were also performed.

Some patients were too young to accept the ERG examination. Some patients refused to accept the ERG detection, So we remove the ERG examination from the experiment.

Whole-body physical examinations

The whole-body examination included routine blood and urine, and liver, kidney, and immune function tests. The venous blood of patients was collected and cluster of differentiation (CD) human T lymphocyte subsets CD3+, CD3+ /CD4+, CD3+/CD8+, and the neutralizing antibody were analyzed by the Department of Laboratory Medicine, Tongji Hospital, Huazhong University of Science and Technology . In addition, we determined the concentration of serum AAV2, ND4, and interferon (IFN)-gamma of the patients via ELISA .

Oral prednisolone

Patients were given a 9-week course of oral prednisolone. This regimen consisted of 0. 5 mg/kg/day for the week before administration of the vector, 1 mg/kg/day during the first week afterward, 0.5 mg/kg/day for the second and third week, 0.25 mg/kg/day

for the fourth and fifth week, and 0.125 mg/kg/day during the last three of the nine weeks. To prevent potentially severe adverse events during and after treatment, we established preventative measures for patients for whom emergencies were a possibility.

Intravitreal injection

Injecting drug: AAV2/2-ND4

Injecting dose: The dose was 5 × 109 vg/0.05 mL for patients younger than 12 years old, and 1 × 1010 vg/0.05 mL for patients older than 12 years old.

In order to improve the therapeutic effect, We increase the injection dose from 0.05 mL to 0.1 mL, but the wall of eyeball in young patients(younger than 12 years old) were very thin, AAV-ND4 refluxed out from the injection site after injection 0.1ml AAV-ND4, So the dose was 5 × 10^9 vg/0.05 mL for patients younger than 12 years old, and 1 × 10^{10} vg/0.05 mL for patients older than 12 years old.

Procedure: The surgery was carried out in the operating room. Tropicamide eye drops (0.5%) were administrated 3 times, 30 minutes before surgery to enlarge the pupils. After pupil enlargement the patients lay supine on the operating table and 0.1% oxybuprocaine hydrochloride eye drops were used to anaesthetize the appropriate eye. The conjunctival sac was washed with 0.3% norfloxacin eye drops. A 1 mL syringe was used, and the injecting pinhead was 30G. The injection site was 3.5 mm away from the temporal limbus. The injecting pinhead was inserted into the conjunctiva, slid for 3 mm along the subconjunctiva, and then entered vertically into the vitreous body. When the pinhead was visible through the pupil, rAAV2-ND4 was injected. The needle was removed from the vitreous cavity. The injection site was pressed with a cotton swab after the intravitreal injection. The injected eye was covered with gauze, and the patient was allowed to lie on the operating table for 30 minutes.

Clinical observation

At months 1, 3, and 6 after surgery whole-body examinations were repeated, and at months 1, 3, 6, after surgery ophthalmologic examinations were repeated.

5. Risks and Benefits to participants and community

Risks

1) Accidents related to anesthesia, such as drug allergy and allergic shock.

2) The oculocardiac reflex may be induced when the eyeball is pressed and the patient's life may be endangered;

3) The lens may be injured during the surgery, and cataract may be a complication;

4) Retinal detachment;

5) Postsurgery infection may affect the healing of the wound;

6) Postsurgical endophthalmitis may be a complication;

7) Eyesight may not improve after surgery;

8) Other potential risks include an immune rejection response, or impairment of liver function, kidney function and functions of other organs which may induce cancer and even endanger life;

9) Prednisone will be administrated orally for eight weeks in the present study, which often leads to upset stomach, increased blood pressure, rash, or urticaria.

10) Other potential unexpected or unpreventable complications.

Benefits to participants:

4、Accept the AAV2-ND4 Treatment free，

5、Ocular and systemic examination free at preoperative and postoperative.

6、Possible to improve vision.

6. Selection and Withdrawal of Subjects

Your participation in the study is completely dependent on your willingness. You can refuse to participate in the study, or, you can also withdraw from the study at any time in the course of research. Such a decision will not affect your relationship with the doctor, nor will it affect your medical or other benefits in any way.

To ensure your maximal benefits, if necessary the doctor or researcher may suspend your participation in the current study at any time during research.

If you withdraw from the current study for any reason, you probably will be asked questions relevant to your medication. If the doctor thinks it is necessary, you will be

asked to accept a laboratory test and physical examination.

7. Treatment of Subjects

The treatment to be administered, including the dose, the dosing schedule, theroute/mode of administration, and the treatment periods, including follow-up: See above.

Procedures to monitor subject compliance: All interventions will be conducted by study personnel; the only subject compliance required is to abide by scheduled visits.

8. Assessment of Efficacy

Visual acuity tests were conducted by an ophthalmologist. The eye chart was a 2.5-m standard ETDRS charts (Star Kang Medical Technology Co., Ltd. Wen Zhou China). We used two different eye charts to check patient's both eyes respectively. The patients were examined repeatedly to confirm their visual improvements, including tests at a different hospital performed by a completely uninformed oculist.

VEP, the function of the optic nerve was examined; primarily the value of the P_{100} wave was determined with a DV-100 (Shanghai Dikon Medical). The tested eyes were optically corrected, and the binocular viewing condition was adopted. The data acquisition and analysis were implemented with a connected computer. Check-reversal amplitude was the difference between the first major positive peak near 100 ms (P_{100}) and the preceding negative peak. The P_{100} amplitude was recorded. Main outcome measures included a latency period of the P_{100} wave and latency period of the reaction capacity of optic nerve conduction signals from the eye to the brain (the value <105 ms regarded as normal).

A Humphrey field analyzer (Carl Zeiss 740i, Carl Zeiss, Shanghai) was used for the visual field test. The testing procedure included the 30-2 central threshold test and SITA fast. Major record parameters were VFI, MD and PSD.

Electroretinogram(ERG)： Some patients were too young to accept the ERG examination. Some patients refused to accept the ERG detection, So we remove the ERG examination from the experiment.

OCT was examined with a Spectralis® HRA + OCT (Heidelberg Engineering, Heidelberg, Germany), and the retinal nerve fiber layer (RNFL) thickness of four quadrants (bottom, up, left and right quadrants) of the retinal vessel was analyzed. All data of OCT were automatically calculated using the existing software.

The above examinations were conducted by two technicians of the Ophthalmology Department.

Increased ocular clinical observation time from six months to nine months.

9. Assessment of Safety

The tonometer used for determining intraocular pressure was a TOPCON-CT-80 Computerized Auto Tonometer (TOPCOCN, Tokyo, Japan). The mean value of three measurements was used.

The fundus (retinal) photography used the NIDEK Autofocus Fundus Camera, AFC-230 (Nidek, Japan).

Whole-body physical examinations： Whole-body physical examinations consisted of routine blood and urine, and liver, kidney, and immune function tests analyzed by the Laboratory Department of Tongji Hospital. The specific examinations were those that are routine at Tongji Hospital.

Routine blood test included 24 items, such as white blood cell count and hemoglobin et al. The routine urine test included 24 items such as red blood cells, white blood cells, and bilirubin in urine et al. Liver and kidney function tests covered 11 items, including alanine aminotransferase, aspartate aminotransferase, and creatinine et al. The immune function test consisted of IgA, IgG, IgM, complement 3 and complement 4. The blood sample was sent to the Laboratory Department of our hospital. The tests for human T lymphocyte subsets CD3+, CD3+/CD4+ and CD3+/CD8+ were conducted by the Central Laboratory of Tongji Hospital. Venous blood from the patients was collected and sent to the Central Laboratory of Tongji Hospital and tested by the staff .

Neutralizing antibody assay： To detect neutralizing antibodies to AAV2, we incubated 1:20, 1:60, 1:180, 1:540, and 1:1620 mouse serum samples with 10^8 vg

AAV2-GFP in 25 μL of PBS for 2 h at 4 °C. This mix was added to each well containing HEK293 cells grown in 6-well plates (to achieve a multiplicity of infection of 1000). The cells were grown at 37 ℃ in 5% CO_2, in Dulbecco's Modified Eagle's Medium (HyClone, Logan, UT) containing 5% FBS (HyClone). Green fluorescent protein expression was evaluated 48 h after infection by flow cytometry (BD Biosciences, NJ, USA). The percentage of inhibition was calculated with no-antibody control samples as a reference. Every experiment was repeated three times.

The levels of AAV2, IFN-γ and ND4 protein determined by ELISA assay： Serum samples from the nine patients were obtained preoperatively and 1, 3, and 6 months after intravitreal injection, and screened by ELISA for immunoreactivity to AAV2, IFN-γ and ND4 protein. The ELISA kits for AAV2, IFN-γ and ND4 were purchased from BlueGene Biotech. Shanghai, China. ELISA was performed according to the manufacturer's protocol.

10. Statistics METHODS

Determination of sample size

We chosed nine patients of LHON to received a single-dose intravitreal injection of rAAV2-ND4. 3 patients received gene therapy in August-September of 2011 as the first group of patients. 6 Patients received gene therapy in October-December of 2012 as the second group of patients.

Statistical analysis

A single statistical analysis will be performed at the end of the study. The data are expressed as mean ± standard error. Record the value of treated eyes, including: BCVA in logMAR , visual field record parameters were VFI, MD and PSD., VEP record parameters were the latency period and the amplitudes of the P100 wave , thickness of the retinal nerve fiber layer, neutralizing antibody, and the concentrations of serum AAV2, ND4, and IFN-gamma were analyzed using the paired t-test. A probability (P) value of less than 0.05 was considered statistically significant.

In the course of the study, We found that the sight of non-treated eyes

has improved,So we Increased statistical analysis about non-treated eye sight , Statistical methods are same of the treated eyes.

Compared visual acuity of treated eyes and non-treated eyes at before treatment, 1, 3, 6, 9 month after treatment.and used the paired t-test. A probability (P) value of less than 0.05 was considered statistically significant.

We didn’t record and analysis of ERG data.

Baseline

The data of treated yees at before treatment as baseline value, including BCVA, VFI, concentrations of serum AAV2, ND4, et al, These data were compared with month 1,3,6 after treatment of treated eyes.

12. ETHICS

Ethics review

The final study protocol, including the final version of the Subject Information and Consent Forms, must be approved in writing by an Independent Ethics Committee (IEC). The Principle Investigator (Clinical Trial Manager) is responsible for informing the IEC of any serious adverse events (SAE) and amendment to the protocol as per regulatory requirement.

Ethical conduct of the study

The study will be performed in accordance with the ethical principles in the Declaration of Helsinki , and that are consistent with Good Clinical Practice and applicable regulatory requirements.

Subject information and consent

The Investigator will ensure that the subject is given full and adequate oral and written information about the nature, purpose, possible risk and benefit of the study. Subjects must also be notified that they are free to discontinue from the study at any time. The subject should be given the opportunity to ask questions and allowed time to consider the information provided. The subject’s signed and dated informed consent must be obtained before conducting any study specific procedure. The investigator must store the original, signed Subject Informed Consent Form and a copy must be given to the subject.

11. STUDY TIME TABLE AND TERMINATION

First group of patients (3 patients)

First subject in	August	2011
Last patient out	March	2012
Clean File	March	2012
Study Report	April	2012

Second group of patients. (6 patients)

First subject in	October	2012
Last patient out	August	2013
Clean File	September	2013
Study Report	October	2013

12. References

1 Y. Mashima, K. Kigasawa, M.Wakakura, et al. Do idebenone and vitamin therapy shorten the time to achieve visual recovery in Leber hereditary optic neuropathy? Journal of Neuro-Ophthalmology. 2000; 20: 166-170.

2 V. Carelli, P. Barboni, A. Zacchini, et al. Leber’s hereditary optic neuropathy (LHON) with 14484/ND6 mutation in a north African patient. Journal of the Neurological Sciences. 1998;160:183-188.

3 Barnils N, Mesa E, Muñoz S, et al. Response to idebenone and multivitamin therapy in Leber’s hereditary optic neuropathy. Archivos de la Sociedad Espanola de Oftalmologia. 2007; 82: 377-380.

4 Angebault C, Gueguen N, Desquiret-Dumas V, et al. Idebenone increases mitochondrial complex I activity in fibroblasts from LHON patients while producing contradictory effects on respiration.BMC Res Notes. 2011, 22;4:557.

5 Klopstock T, Yu-Wai-Man P, Dimitriadis K, et al. A randomized placebo-controlled trial of idebenone in Leber's hereditary optic neuropathy. Brain. 2011; 134:2677-2686.

6 Carelli V, La Morgia C, Valentino ML, et al. Idebenone treatment in Leber's hereditary optic neuropathy. Brain. 2011; 134:e188.

7 Sadun AA, Chicani CF, Ross-Cisneros FN, et al. Effect of EPI-743 on the clinical course of the mitochondrial disease Leber hereditary optic neuropathy. Arch Neurol. 2012; 69:331-338.
