# Supplementary material for: Efficacy and Safety of rAAV2-ND4 Treatment for Leber’s Hereditary Optic Neuropathy
Source: Sci Rep. 2016 Feb 19;6:21587. doi: 10.1038/srep21587 (PMC4759604; doi:10.1038/srep21587)
Supplement: Supplementary Information 1 [file srep21587-s2.pdf]

Efficacy and Safety of rAAV2-ND4 Treatment for Leber's Hereditary Optic Neuropathy

Xing Wan[1], Han Pei[1], Min-jian Zhao[2], Shuo Yang[1], Wei-Kun Hu[1], Heng He[1], Si-qi Ma[1], Ge Zhang[3], Xiao-yan Dong[4], Chen Chen[5], Dao-wen Wang[5] & Bin Li[1]*

Department of Ophthalmology, Tongji Hospital, Tongji Medical College, Huazhong University of Science and Technology, Wuhan, China, [2]Department of Oncology, Central Hospital, Ezhou City, China, [3]Department of Microbial and Biochemical Pharmacy, School of Pharmaceutical Sciences, Sun Yat-sen University, Guangzhou, China, [4]Beijing FivePlus Molecular Medicine Institute Co. Ltd., Beijing, China, [5]Center Genetic Diagnosis, Tongji Hospital, Tongji Medical College, Huazhong University of Science and Technology, Wuhan, China

***Correspondence to:** Professor Bin Li (libin-12@163.com)

Xing Wan, Han Pei, Min-jian Zhao and Shuo Yang contributed equally to this article.

Ophthalmologic examination results. In a preliminary trial that included three patients, the IOP and the anterior and posterior segments of the eye were not changed from before to after intravitreal injection. In this study, which included nine patients, there were no significant changes in the posterior segments of the eye.

There were no significant changes in VEP, P_{100} wave latency, and amplitude from before to 9 months after intravitreal injection, but the trend was of an improvement (Supplementary Fig. S5).

OCT indicated that there was no significant change in the optic nerve fibre layer thickness of the nine patients in the superior, nasal, inferior, and temporal quadrants after intravitreal injection (Supplementary Fig. S6).

Systemic physical examinations. Before gene therapy, all patients underwent detailed systemic physical and ophthalmologic examinations. Systemic physical examinations consisted of routine blood and urine, and liver, kidney, and immune function tests analysed by the Laboratory Department of Tongji Hospital. The specific examinations were those that are routine at Tongji Hospital. Routine blood test included 24 items, including white blood cell count and haemoglobin. The routine urine test included 24 items, including presence of red blood cells, white blood cells, and bilirubin. Liver and kidney function tests covered 11 items, including alanine aminotransferase, aspartate aminotransferase, and creatinine. The immune function test consisted of IgA, IgG, IgM, complement 3, and complement 4. The blood sample was sent to the Laboratory Department of our hospital. The tests for human T

lymphocyte subsets CD3+, CD3+/CD4+ and CD3+/CD8+ were conducted by the Central Laboratory of Tongji Hospital. Venous blood from the patients was collected and sent to the Central Laboratory of Tongji Hospital and tested by the staff (Supplementary Fig. S1).

The third part of the physical exam was a neutralizing antibody assay performed by using flow cytometry, as well as the determination of serum concentration of ND4, AAV, and IFN-γ of patients evaluated by ELISA.

Neutralizing antibody assay. To detect neutralizing antibodies to AAV2, we incubated 1:20, 1:60, 1:180, 1:540, and 1:1620 human serum samples with 10^8 vg AAV2-GFP in 25 μL of PBS for 2 h at 4°C. This mix was added to each well containing HEK293 cells grown in 6-well plates (to achieve a multiplicity of infection of 1000). The cells were grown at 37°C in 5% CO_2, in Dulbecco's Modified Eagle's Medium (HyClone, Logan, UT) containing 5% fetal bovine serum (FBS; HyClone). Green fluorescent protein (GFP) expression was evaluated 48 h after infection by flow cytometry (BD Biosciences, NJ, USA). The percentage of inhibition was calculated with no-antibody control samples as a reference. Each experiment was repeated three times.

AAV2, IFN-γ, and ND4 protein levels determined by ELISA. Serum samples from the nine patients were obtained preoperatively and 1, 3, and 6 months after intravitreal injection, and screened by ELISA for immunoreactivity to AAV2, IFN-γ and ND4 protein. The ELISA kits for AAV2, IFN-γ and ND4 were purchased from

BlueGene Biotech. Shanghai, China. ELISA was performed according to the manufacturer's protocol.

Mean serum ND4 concentrations (ng/mL) were 1.34 ± 0.66, 1.33 ± 0.69, 1.25 ± 0.48, and 1.45 ± 0.38 before and 1, 3, and 6 months after intravitreal injection, respectively; the differences were not statistically significant. Mean serum AAV2 concentrations (ng/mL) were 8.35 ± 7.37, 7.42 ± 4.17, 6.67 ± 4.27, and 9.18 ± 6.32 before and 1, 3, and 6 months after intravitreal injection, respectively; the differences were not statistically significant. Mean serum IFN-γ concentrations (ng/mL) were 106.54 ± 37.53, 87.28 ± 13.85, 95.90 ± 30.08, and 83.96 ± 20.92 before and 1, 3, and 6 months after intravitreal injection (Supplementary Fig. S2).

Supplementary figures

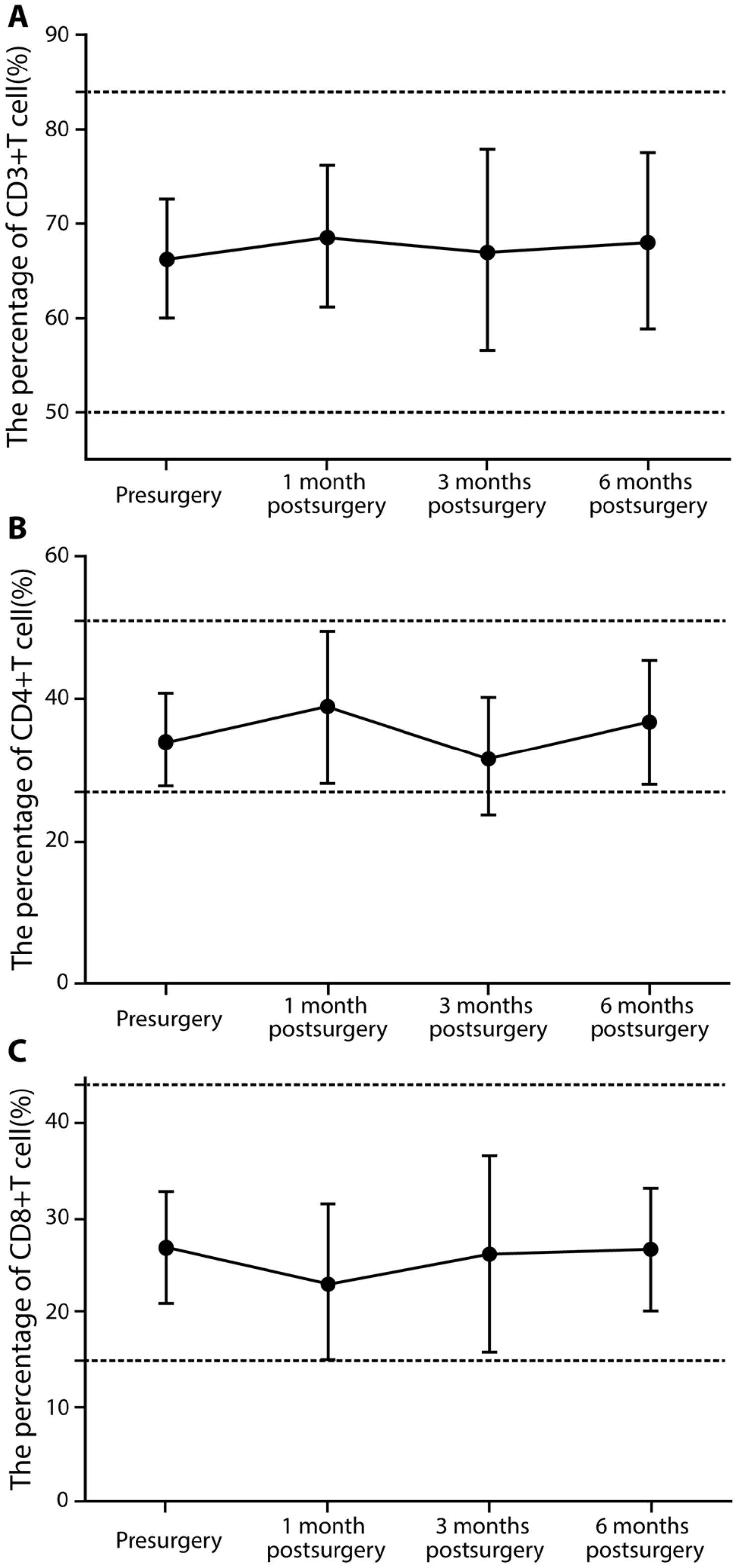

Fig. S1. Percentage of CD3+, CD4+, and CD8+ cells before and 1, 3, and 6 months after intravitreal injection. (**A**) CD3+; (**B**) CD4+; and (**C**) CD8+ cells (*N* = 9).

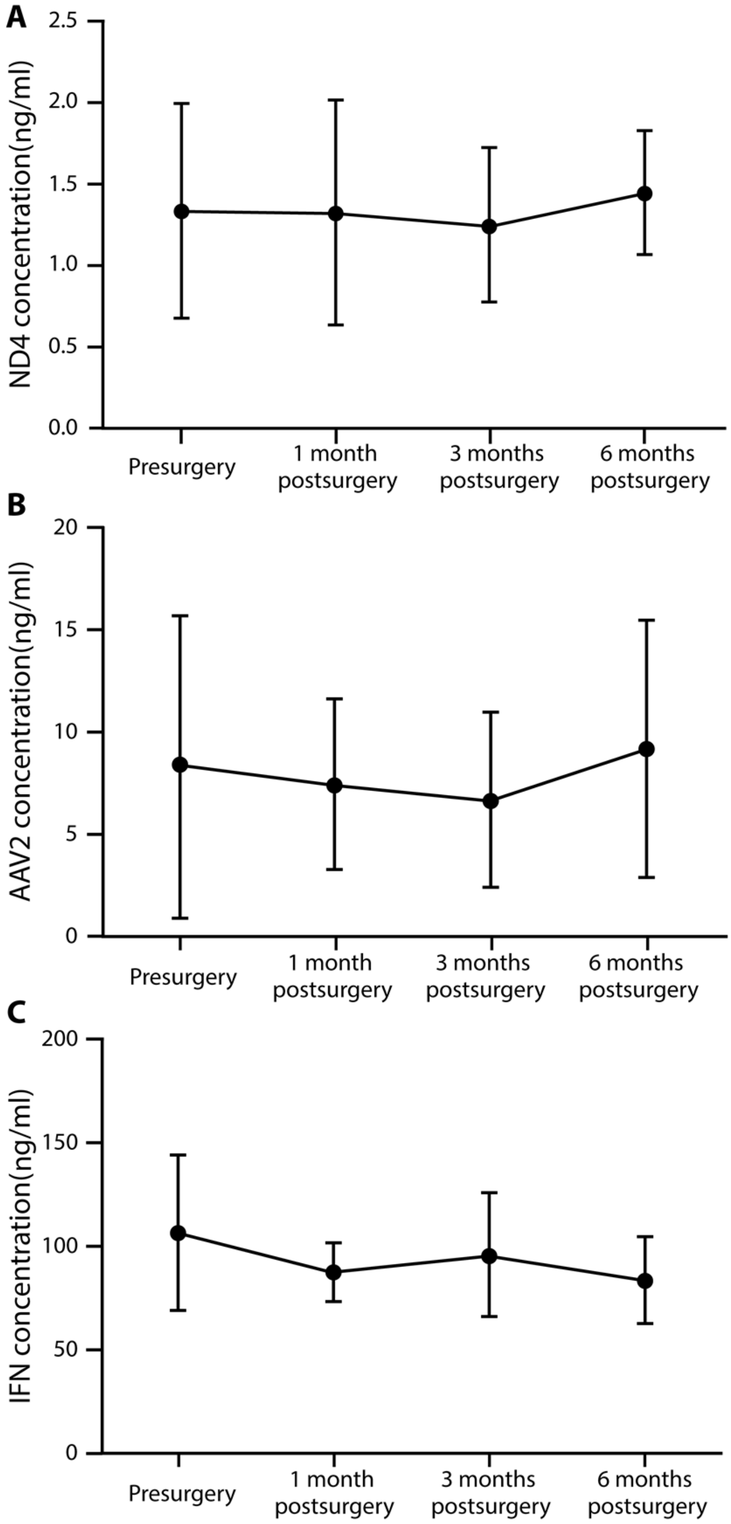

Fig. S2. Serum concentrations of ND4, AAV2, and IFN-γ before and 1, 3, and 6 months after intravitreal injection. The only significant change (an increase) was IFN-γ level at 6 months (*P* = 0.04).

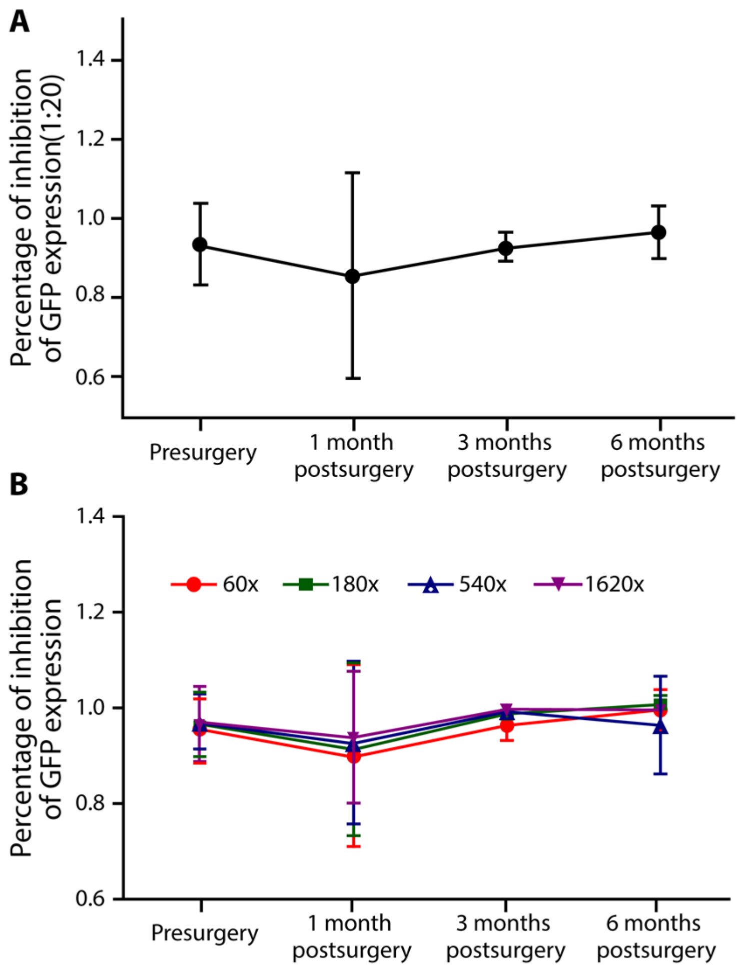

Fig. S3. Anti-AAV2-neutralizing antibody assay. Before and at months 1 and 6 after intravitreal injection, the neutralizing antibody assay of all patients was negative. However, it was positive 3 months after intravitreal injection. (**A**) Percentage of inhibition of GFP expression (1:20). (**B**) Percentage of inhibition of GFP expression (1:60, 1:180, 1:540, and 1:1620). Positive means that the anti-AAV2 neutralizing antibody assay was significantly different compared to serum-free medium with 1:20 serum concentrations.

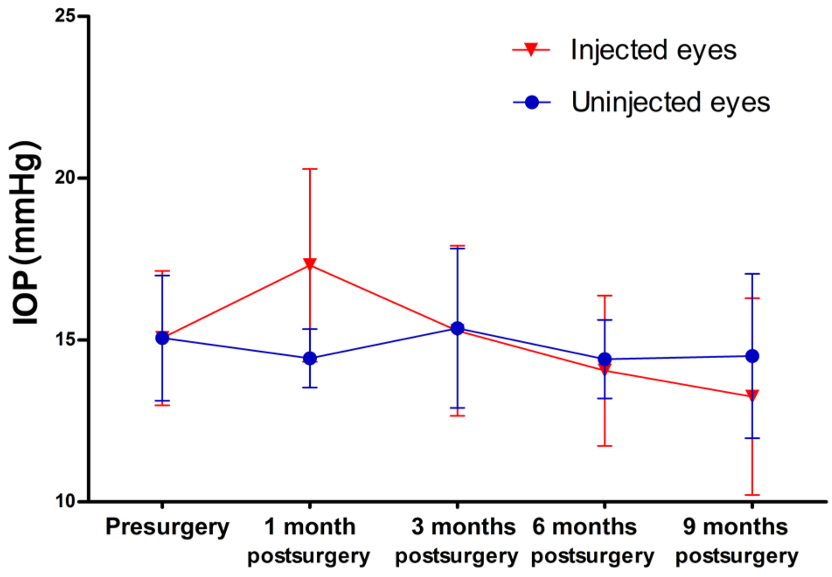

Fig. S4. IOP (mmHg) before and 1, 3, and 6 months after intravitreal injection.

There were no significant differences in IOP in the nine patients.

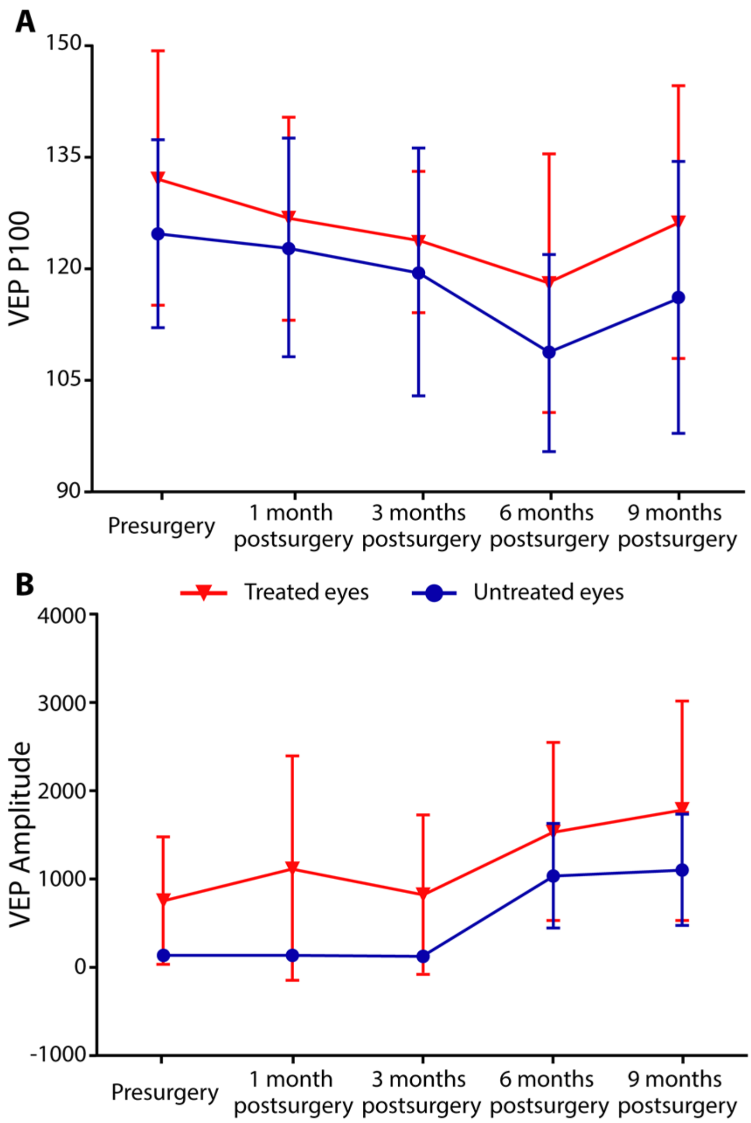

Fig. S5. Oscillogram of pattern-reversal visual evoked potential (PR-VEP) and P_{100} amplitude in PR-VEP. (**A**) Latency period of the P_{100} wave and (**B**) P_{100} amplitude of injected and uninjected eyes in the nine patients.

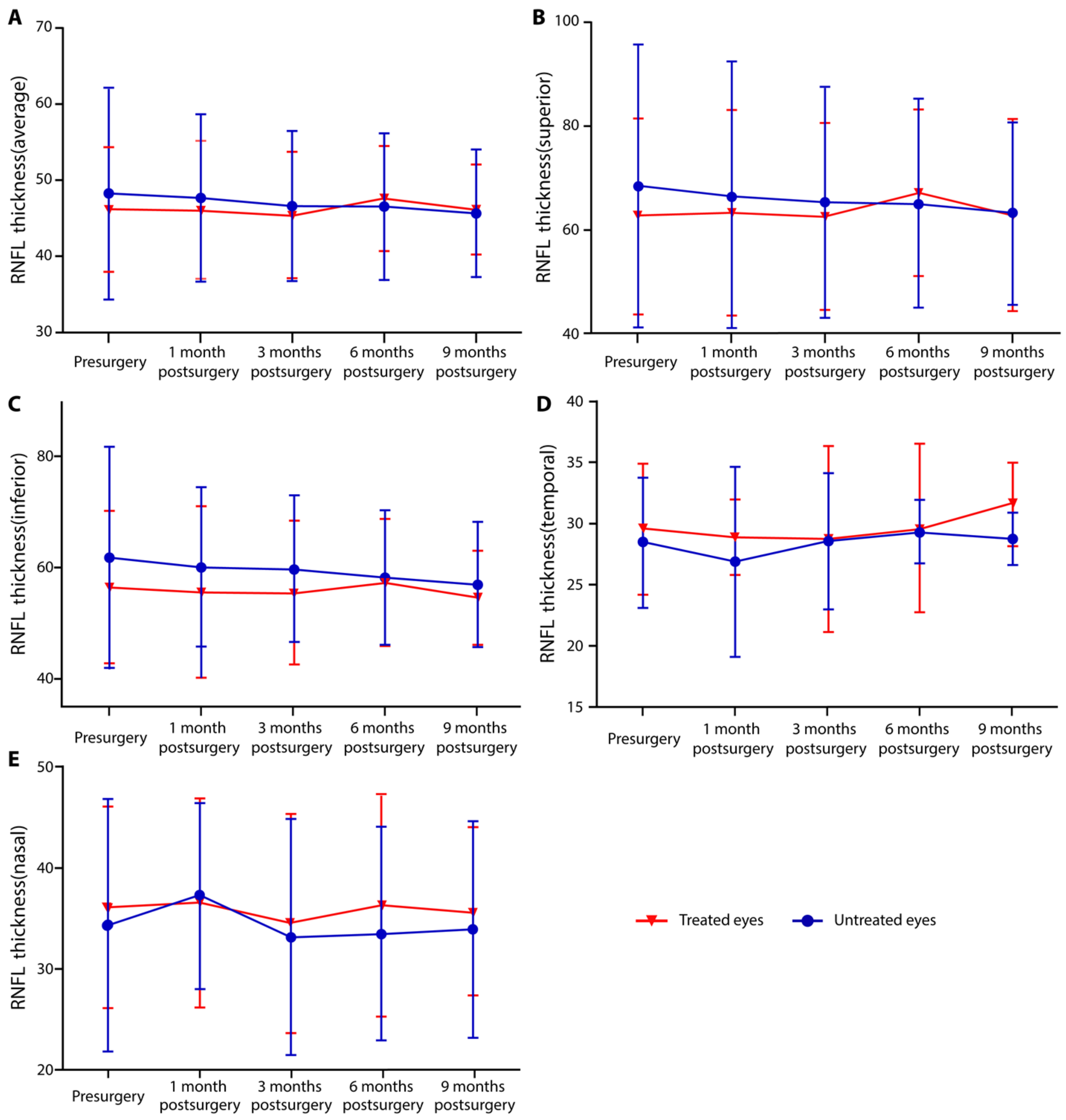

Fig. S6. RNFL measured by OCT before and 1, 3, and 6 months after intravitreal injection. (**A**) Average thickness of the RNFL; (**B**) superior thickness of the RNFL; (**C**) inferior thickness of the RNFL; (**D**) temporal thickness of the RNFL; and (**E**) nasal thickness of the RNFL (*N* = 9). There were no significant differences in RNFL in the nine patients.
